# ROB-MEN: a tool to assess risk of bias due to missing evidence in network meta-analysis

**DOI:** 10.1186/s12916-021-02166-3

**Published:** 2021-11-23

**Authors:** Virginia Chiocchia, Adriani Nikolakopoulou, Julian P. T. Higgins, Matthew J. Page, Theodoros Papakonstantinou, Andrea Cipriani, Toshi A. Furukawa, George C. M. Siontis, Matthias Egger, Georgia Salanti

**Affiliations:** 1 Institute of Social and Preventive Medicine, University of Bern, Bern, Switzerland; 2 Graduate School for Health Sciences, University of Bern, Bern, Switzerland; 3 Institute of Medical Biometry and Statistics, Faculty of Medicine and Medical Center, University of Freiburg, Freiburg im Breisgau, Germany; 4 Population Health Sciences, Bristol Medical School, University of Bristol, Bristol, UK; 5 School of Public Health and Preventive Medicine, Monash University, Melbourne, Australia; 6 Department of Psychiatry, University of Oxford, Oxford, UK; 7 Oxford Health NHS Foundation Trust, Warneford Hospital, Oxford, UK; 8 Department of Health Promotion and Human Behavior, Kyoto University Graduate School of Medicine and School of Public Health, Kyoto, Japan; 9 Department of Cardiology, Bern University Hospital, Inselspital, Bern, Switzerland; 10 Centre for Infectious Disease Epidemiology and Research, University of Cape Town, Cape Town, South Africa

**Keywords:** Risk of bias, Missing evidence, Network meta-analysis, Evidence synthesis, Publication bias, Selective outcome reporting, Reporting bias

## Abstract

**Background:**

Selective outcome reporting and publication bias threaten the validity of systematic reviews and meta-analyses and can affect clinical decision-making. A rigorous method to evaluate the impact of this bias on the results of network meta-analyses of interventions is lacking. We present a tool to assess the Risk Of Bias due to Missing Evidence in Network meta-analysis (ROB-MEN).

**Methods:**

ROB-MEN first evaluates the risk of bias due to missing evidence for each of the possible pairwise comparison that can be made between the interventions in the network. This step considers possible bias due to the presence of studies with unavailable results (*within-study assessment of bias*) and the potential for unpublished studies (*across-study assessment of bias*). The second step combines the judgements about the risk of bias due to missing evidence in pairwise comparisons with (i) the contribution of direct comparisons to the network meta-analysis estimates, (ii) possible small-study effects evaluated by network meta-regression, and (iii) any bias from unobserved comparisons. Then, a level of “low risk”, “some concerns”, or “high risk” for the bias due to missing evidence is assigned to each estimate, which is our tool’s final output.

**Results:**

We describe the methodology of ROB-MEN step-by-step using an illustrative example from a published NMA of non-diagnostic modalities for the detection of coronary artery disease in patients with low risk acute coronary syndrome. We also report a full application of the tool on a larger and more complex published network of 18 drugs from head-to-head studies for the acute treatment of adults with major depressive disorder.

**Conclusions:**

ROB-MEN is the first tool for evaluating the risk of bias due to missing evidence in network meta-analysis and applies to networks of all sizes and geometry. The use of ROB-MEN is facilitated by an R Shiny web application that produces the Pairwise Comparisons and ROB-MEN Table and is incorporated in the reporting bias domain of the CINeMA framework and software.

**Supplementary Information:**

The online version contains supplementary material available at 10.1186/s12916-021-02166-3.

## Background

A challenging issue in evidence-based medicine is the bias introduced by the selective non-reporting of primary studies or results. Failure to report all findings can lead to results being missing from a meta-analysis. Either a whole study may remain unpublished, commonly referred to as ‘publication bias’, or specific results may not be reported in a publication, usually referred to as ‘selective outcome reporting bias’ or ‘selective non-reporting of results’.

Several methods are available to investigate such bias in pairwise meta-analysis [1]. These include generic approaches, for example, comparisons of study protocols with published reports and comparison of results obtained from published versus unpublished sources, as well as statistical methods (e.g. funnel plots [2–4], tests for small-study effects [2, 5–7] and selection models [8, 9]). Recently, a tool to evaluate Risk Of Bias due to Missing Evidence (ROB-ME) integrated these approaches into an overall assessment of the risk of bias due to missing evidence in pairwise meta-analysis [10].

Network meta-analysis extends pairwise meta-analysis to enable multiple treatments comparison by combining direct and indirect evidence within a network of randomised trials or other comparative studies. Several of the numerical approaches to evaluate bias developed for pairwise meta-analysis have been adapted to the network meta-analysis setting [11–15]. Still, a rigorous methodology for assessing the risk of bias due to missing results in network meta-analysis estimates is currently lacking.

To address this gap, we developed the Risk Of Bias due to Missing Evidence in Network meta-analysis (ROB-MEN) tool, which incorporates qualitative and quantitative methods. We assume that investigators assembled studies into a coherent network according to a pre-specified protocol, checked the assumptions and deemed them plausible and used appropriate statistical methods to obtain relative treatment effects for pairs of interventions. Then, ROB-MEN can be used to assess the risk of bias due to missing evidence in each of the relative treatment effects estimated in network meta-analysis. We illustrate the ROB-MEN approach step by step using a network meta-analysis of non-invasive diagnostic tests for coronary artery disease [16]. We also report an application of the tool to a network of 18 antidepressants from head-to-head studies [17].

## Methods

The ROB-MEN tool was developed between April and November 2020 within the CINeMA framework to evaluate confidence in results from network meta-analysis [18, 19]. The authors are epidemiologists, statisticians, systematic reviewers, trialists, and health services researchers, many of whom are involved with Cochrane systematic reviews, methods groups, and training events. The initial proposal drew on existing methods for assessing selective outcome reporting bias [20] and publication bias [2, 5, 8] in pairwise meta-analysis, as summarised in the Cochrane Handbook for Systematic Reviews of Interventions [1]. A draft tool was developed in line with the preliminary version of the ROB-ME tool [10] and presented to all co-authors. Improvements and modifications were informed by relevant methodological literature, previously published tools for assessing methodological quality of meta-analyses and by the authors’ experience of developing tools to assess the risk of bias in randomised and non-randomised studies, and systematic reviews [21, 22]. The group met several times to discuss the approach and agreed on the tool’s structure, content, and step-wise application. An R Shiny web application to facilitate the implementation of ROB-MEN for the users was developed alongside the tool’s conceptual framework by two of the co-authors and checked by the whole group. Refinements were made following feedback received also from training and research events.

**Fig. 1:**
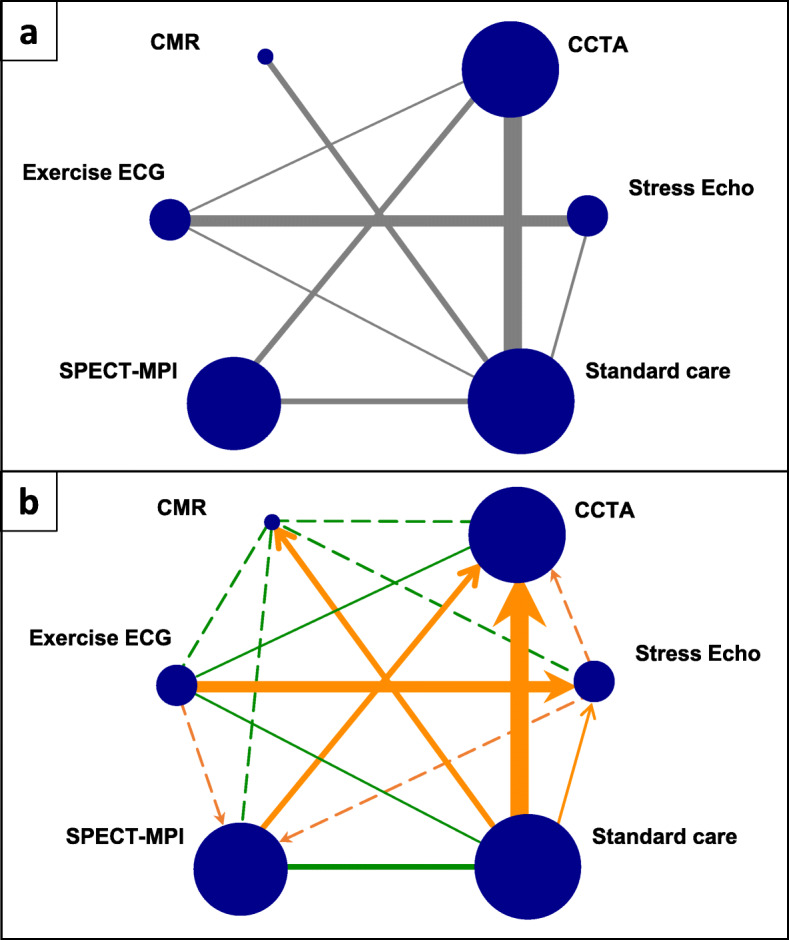
Network plots of network meta-analysis of non-invasive diagnostic modalities for detecting coronary artery disease. **a** Standard network plot. **b** Network graph showing risk of bias assessment for pairwise comparisons. Sizes of solid lines and nodes are proportional to number of studies in each comparison and total sample size for each treatment, respectively. Solid lines represent the observed direct comparisons, dotted lines represent unobserved comparisons between interventions. Green indicates *no bias detected*, orange indicates *suspected bias favouring the treatment indicated by the arrow*. ECG: electrocardiogram; CCTA: coronary computed tomographic angiography; CMR: cardiovascular magnetic resonance; SPECT-MPI: single-photon emission computed tomography-myocardial perfusion imaging; Stress Echo: stress echocardiography

We outline the methodology using the example of a network of randomised controlled trials comparing non-invasive diagnostic strategies for the detection of coronary artery disease in patients presenting with symptoms suggestive of an acute coronary syndrome [16]. The outcome of interest is referral to coronary angiography, for which the network included 18 trials comparing exercise electrocardiogram (ECG), single-photon emission computed tomography-myocardial perfusion imaging (SPECT-MPI), coronary computed tomographic angiography (CCTA), cardiovascular magnetic resonance (CMR), stress echocardiography (Stress echo), and standard care. Standard care was based on the discretion of the clinicians or local diagnostic strategies. The network graph is shown in Fig. 1a, and a summary of the network meta-analysis methods and results is available in Additional file 1.

### Overview of ROB-MEN

In ROB-MEN, ‘bias due to missing evidence’ refers to bias arising when some study results are unavailable because of their results. This situation may, for example, arise because of non-significant *p*-values, small magnitudes of effect, or harmful effects. It can be due to two types of missing evidence, as described in the recently developed ROB-ME tool [10]: (i) the selective reporting of results within studies that are published or otherwise known to exist, called “*within-study assessment of bias*” in the tool; (ii) studies that remain entirely unpublished and are not known to exist, referred to as “*across-study assessment of bias*” (see also the glossary in Table 1).

**Table 1:** Glossary of terms

**Pairwise comparisons:** All treatment comparisons in the network irrespective of the availability of data. A network with T treatments has T(T-1)/2 pairwise comparisons. Depending on whether there are studies reporting the studied outcome, the pairwise comparisons can be distinguished into *observed for this outcome, observed for other outcomes,* and *unobserved*. **Direct evidence:** The evidence available (statistical information derived from data) about a pairwise comparison that is available from direct, within study information about that comparison. **Indirect evidence:** The evidence available (statistical information derived from data) about a pairwise comparison that is *not* available from within study information, i.e. is obtained indirectly via a common comparator or chain of comparisons. **‘Only direct’ estimate:** Relative treatment effect estimated in an network meta analysis that is derived only from direct evidence. **‘Only indirect’ estimate:** Relative treatment effect estimated in an network meta analysis that is derived only from indirect evidence. **Mixed estimate:** Relative treatment effect estimated in an network meta analysis that is derived from both direct and indirect evidence. **Network meta-analysis estimates:** Estimates of relative treatment effects derived from network meta analysis; these can be distinguished into ‘Only direct’, ‘Only indirect’ and Mixed estimates. **Within-study assessment of bias due to missing evidence:** Bias arising from missing results due to selective outcome reporting i.e. results being reported, but not others, within studies published or otherwise known to exist. **Across-study assessment of bias due to missing evidence:** Bias introduced from missing studies because they are entirely unpublished i.e. not known to exist.	

In network meta-analysis, estimates of treatment effects are derived by combining direct and indirect evidence. Direct evidence refers to evidence about pairs of treatments that have been directly compared within studies (e.g. the 8 pairwise comparisons with data shown in Fig. 1a). Indirect evidence refers to evidence on pairs of treatments that is “indirectly” derived from the sources of direct evidence via a common comparator or chain of comparisons (Table 1). In ROB-MEN, we first evaluate the likely risk of bias due to missing evidence for each pairwise comparison between the interventions of interest, irrespective of the availability of direct evidence (Fig. 1b). We then consider the risk of bias from pairwise comparisons and their contribution to each estimate [23] with the additional risk of bias from indirect comparisons and any evidence of small-study effects to evaluate the overall risk of bias due to missing evidence in each network meta-analysis estimate.

**Fig. 2:**
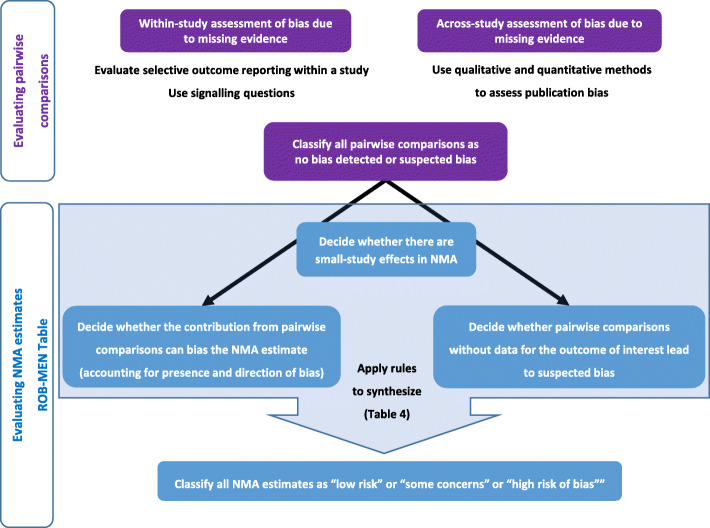
Overview of the ROB-MEN process

Two tables that record the assessments for each pairwise comparison and each estimate are at the tool’s core: the *Pairwise Comparisons Table* and the *ROB-MEN Table* (see Tables 2 and 3 for examples). Both tables are completed separately for each outcome in the review. The Pairwise Comparisons Table facilitates the assessments in the ROB-MEN Table. The output of the Pairwise Comparisons Table provides judgement on possible bias due to missing evidence for each of the possible comparisons made from the interventions in the network. The ROB-MEN Table is the main output of the tool. It combines the information from the Pairwise Comparisons Table with (i) information about the structure and the amount of data in the network and (ii) the potential impact of missing evidence on the network meta-analysis results to reach an overall judgement about the risk of bias for each estimate. Figure 2 summarises the process. An R Shiny web application (https://cinema.ispm.unibe.ch/rob-men/) facilitates the ROB-MEN process, including creating the two core tables, as described in Additional file 2 and Additional file 3 [24].

**Table 2:** Pairwise Comparisons Table for the network of non-invasive diagnostic modalities for detecting coronary artery disease

Column no.	1	2	3	4	5
Pairwise comparisons	No. of studies in each comparison		Within-study assessment of bias	Across-study assessment of bias	Overall bias
	Reporting this outcome (sample size)	Total identified in the SR (total sample size)	Evaluation of selective reporting within studies using signalling questions	Qualitative and quantitative assessment of publication bias	Overall judgement
**Group A: observed for this outcome**					
CCTA vs exercise ECG	1 (562)	1 (562)	No bias detected	No bias detected	No bias detected
CCTA vs SPECT-MPI	2 (1149)	2 (1149)	No bias detected	Suspected bias favouring CCTA	Suspected bias favouring CCTA
CCTA vs standard care	7 (4015)	7 (4015)	No bias detected	Suspected bias favouring CCTA	Suspected bias favouring CCTA
CMR vs standard care	2 (214)	2 (214)	No bias detected	Suspected bias favouring CMR	Suspected bias favouring CMR
Exercise ECG vs standard care	1 (130)	1 (130)	No bias detected	No bias detected	No bias detected
Exercise ECG vs stress echo	4 (1086)	4 (1086)	No bias detected	No bias detected	No bias detected
SPECT-MPI vs standard care	2 (4165)	2 (4165)	No bias detected	No bias detected	No bias detected
Standard care vs stress echo	1 (132)	1 (132)	No bias detected	Suspected bias favouring Stress Echo	Suspected bias favouring Stress Echo
**Group B: observed for other outcomes (no studies)**					
**Group C: Unobserved**					
CCTA vs CMR	0	0	NA	No bias detected	No bias detected
CCTA vs stress echo	0	0	NA	Suspected bias favouring CCTA	Suspected bias favouring CCTA
CMR vs exercise ECG	0	0	NA	No bias detected	No bias detected
CMR vs SPECT-MPI	0	0	NA	No bias detected	No bias detected
CMR vs stress echo	0	0	NA	Suspected bias favouring CMR	Suspected bias favouring CMR
Exercise ECG vs SPECT-MPI	0	0	NA	Suspected bias favouring SPECT-MPI	Suspected bias favouring SPECT-MPI
SPECT-MPI vs stress echo	0	0	NA	No bias detected	No bias detected

CCTA, coronary computed tomographic angiography; CMR, cardiovascular magnetic resonance; ECG, electrocardiogram; Echo, echocardiography; SPECT-MPI, single-photon emission computed tomography-myocardial perfusion imaging; SR, systematic review

**Table 3:**
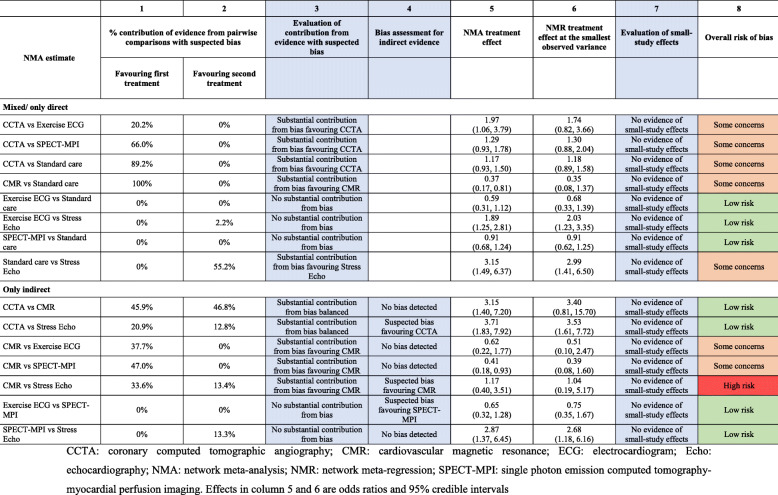
ROB-MEN Table for the network of non-invasive diagnostic modalities for detection of coronary artery disease in patients with low risk acute coronary syndrome

### Risk of bias due to missing evidence in pairwise comparisons

The assessment of bias due to missing evidence in all possible pairwise comparisons follows the ROB-ME tool for pairwise meta-analysis [10]. Like ROB-ME, we consider the studies contributing to the network meta-analysis of the outcome of interest and the studies contributing to networks of other outcomes in a systematic review. Such studies are informative about possible selective non-reporting of the outcome being addressed in the current network meta-analysis. ROB-MEN differs from ROB-ME by considering all possible pairwise comparisons between the interventions in the network. There may be missing evidence for any directly observed comparisons and missing evidence for the indirect comparisons that were *not* observed among the included studies. The possible pairwise comparisons between the interventions involved in the network, that is, all combinations of two treatments, are organised into three groups:

A. “Observed for this outcome”: the comparisons for which there is direct evidence contributing to the network meta-analysis for the current outcome
B. “Observed for other outcomes”: the pairwise comparisons for which there is direct evidence only for other outcomes in the systematic review
C. “Unobserved”: the pairwise comparisons that have not been investigated in any of the identified studies in the systematic review.

These groups constitute the rows of the Pairwise Comparisons Table for a specific outcome. Instructions for filling in the table are summarised in Additional file 2.

For each comparison, the first two columns report the total number of studies with results for the current outcome or any outcome, respectively. In brackets, we enter the total sample size by adding up all participants randomised in the studies investigating the specific comparison for that outcome. By definition, the unobserved comparisons will have zero in both columns. In contrast, those observed for other outcomes will have zero in the first column.

The groups of comparisons are presented in Table 2 for the example of non-invasive diagnostic modalities for the detection of coronary artery disease. Of the possible 15 comparisons, 8 were observed for the outcome of interest. The remaining 7 were unobserved, i.e. not observed for the outcome of interest or any other outcomes.

#### Within-study assessment of bias due to missing evidence

The evaluation of bias due to selective non-reporting of results within studies concerns studies identified for the review but missing from the synthesis. They are known to exist, but the results are unavailable: the studies report on other outcomes than the outcome of interest. The presence of selective non-reporting of results in each study is assessed using study-specific tools such as Step 2 of the ROB-ME tool [10, 20]. Then, the likely impact of the missing results across all studies may be assessed using two signalling questions to reach an overall judgement of *no bias detected* or *suspected bias favouring X* for each comparison (Table 4). The preliminary version of the ROB-ME tool describes various approaches to evaluate the within-study assessment of bias by considering the plausibility of scenarios where study results are or are *not* unavailable because of the *p*-value, magnitude, or direction of the treatment effects [1, 10].

**Table 4:** Signalling questions for the within-study bias assessment of comparisons observed for the outcome of interest or other outcomes

Signalling question	Responses for each comparison (groups A and B only)		
1. Was there any eligible study for which results for the outcome of interest were unavailable, likely because of the *p*-value, magnitude or direction of the result generated?	Yes	Yes	No
2. (If Yes to the previous question) Was the amount of information omitted from the synthesis sufficient to have a notable effect on the magnitude of the synthesised result?	Yes	No	-
Overall judgement	*Suspected bias (favouring X)*	*No bias detected*	*No bias detected*

A thorough within-study assessment of bias due to missing evidence is labour intensive but particularly valuable as the impact of selective non-reporting or under-reporting of results can be quantified more easily than the impact of selective non-publication of an unknown number of studies [1]. However, suppose the number of studies (or the sample size) not reporting the outcome of interest (i.e. the difference between the first two columns in Table 2) is small compared to the number of studies (or the total sample size) reporting the outcome (the first column in Table 2). In that case, the assessment of these few studies is unlikely to affect the judgement from the within-study assessment significantly. Reviewers may then decide to assign *no bias detected* to the relevant comparison without carrying out the assessment. *No bias detected* is also assigned when no study is suspected of selective non-reporting or under-reporting of results for a specific comparison (i.e. the numbers in the first two columns are equal). For the unobserved comparisons, the assessment is not applicable (“NA”, Table 2).

In the example of the non-invasive diagnosis of coronary artery disease, there were no additional studies that did not report results for the outcome of interest. Therefore, we assume that there is no selective outcome reporting bias, and we assign *no bias detected* for the within-study assessment of bias to all observed comparisons. In the ‘14’ section, the within-study assessment of bias is completed using the signalling questions for additional studies not reporting the outcome of interest.

#### Across-study assessment of bias due to missing evidence

This situation refers to studies undertaken but not published, so reviewers are unaware of them. Each comparison is assessed for risk of publication bias using qualitative and quantitative considerations. First, a qualitative judgement is made to assign a level of *no bias detected* or *suspected bias*. Conditions that may indicate bias include:

- Failure to search for unpublished studies and grey literature
- The meta-analysis may be based on a few positive findings on a newly introduced drug as the early evidence likely overestimates efficacy [25]
- Previous evidence may have shown the presence of publication bias for that comparison [26]

Conditions suggesting no bias include data from unpublished studies and agreement of their findings with those of published studies or a tradition of prospective trial registration in the field.

For comparisons with at least 10 studies (in the first column in Table 2), judgements can additionally consider statistical techniques such as contour-enhanced funnel plots [4], meta-regression models and statistical tests for small-study effects [2, 6, 7, 27–29], or selection models for pairwise meta-analysis (e.g. Copas [8]). These can be useful when it is difficult to assess publication bias reliably, e.g. when protocols and records from trial registries were unavailable. The direction of any bias should be noted: it will generally reflect the larger benefits observed in smaller studies.

We implemented the across-study assessment of bias in the network meta-analysis of non-invasive diagnostic tests of coronary artery disease using qualitative considerations (see Additional file 4). None of the comparisons included 10 or more studies and no assessment using graphical or statistical methods was therefore performed. The judgements for all comparisons are reported in Table 2.

#### Overall risk of bias for pairwise comparisons

The last step in the Pairwise Comparisons Table is to combine the levels of risk assigned in the previous steps into a final judgement of *no bias detected* or *suspected bias*. In case of *suspected bias*, the predicted direction of the bias, i.e. which treatment the bias is likely to favour, should also be specified (see Fig. 1). For the unobserved comparisons (group C), the overall risk of bias will be the same as the judgement made for the across-study assessment of bias, as this is the only assessment applicable to these comparisons.

For the comparisons observed for the outcome of interest or other outcomes (group A and B), the overall judgement will consider qualitative assessments for both the within-study and the across-study assessment of bias. The assessment of selective outcome reporting bias (“within-study assessment of bias”) is likely to be the most valuable because its impact can be quantified more easily than that of publication bias (“across-study assessment of bias”). The process of forming a final judgement for each pairwise comparison is illustrated in the flowchart in Additional file 5.

Since there was no within-study assessment of bias for the example of non-invasive diagnosis of coronary artery disease, the overall bias judgement will only consider the across-study assessment of bias. The final overall risk of bias judgements is reported in the Pairwise Comparison Table (Table 2).

### Risk of bias due to missing evidence in network meta-analysis estimates

Once the assessments of overall bias for each pairwise comparison are complete, we integrate them in the assessment of risk of bias for each network estimate in the ROB-MEN Table. We organise the estimates into two groups, “mixed/only direct” and “only indirect”, depending on the type of evidence contributing to each estimate (see Table 1). Here, we describe the detailed steps for filling in the relevant column in the ROB-MEN Table and illustrate them using the network of trials of non-invasive coronary artery disease diagnosis. Instructions are summarised in Additional file 3.

#### Contribution of comparisons with suspected bias to network meta-analysis estimates

The first step is to consider the contribution matrix of the network. The cells of this matrix provide the percentage contribution that each comparison with direct evidence (columns of the matrix) makes to the calculation of the corresponding network meta-analysis relative treatment effect (rows of the matrix) [23]. Additional file 6 shows the contribution matrix for the network of non-invasive diagnosis of coronary artery disease. Each comparison with direct evidence is combined with the risk of bias as judged in the Pairwise Comparisons Table (Table 2). This way, the percentage contribution from direct evidence with suspected bias (reported in the first and second column of the ROB-MEN Table, see Table 3 for example) can be estimated. The evaluation of the contribution from comparisons with suspected bias is reported in the third column. Specifically, the possible levels are:

- *No substantial contribution from bias*: there is no substantial contribution from evidence with bias favouring one of the two treatments;
- *Substantial contribution from bias balanced*: there is a substantial contribution from evidence with suspected bias, but the biases favouring one or the other treatment are balanced and cancel each other out;
- *Substantial contribution from bias favouring X*: there is a substantial contribution from evidence with bias favouring one of the two treatments (say X).

In the non-invasive diagnosis of coronary artery disease network meta-analysis, we considered the contribution from biased evidence as substantially in favour of one treatment if the relative difference between treatments was at least 15%. Among the mixed estimates, five of them have a clear separation of high contribution coming from biased evidence between the two treatments (e.g. CCTA vs SPECT-MPI). Among the indirect estimates, only three estimates showed such clear separation (e.g. CMR vs SPECT-MPI). The relevant bias judgements for this step are in column 3 of the ROB-MEN Table (Table 3).

#### Additional risk of bias for indirect estimates

Indirect relative effects are calculated from sources of direct evidence in the Pairwise Comparisons Table with contributions as shown in the contribution matrix. The absence of direct evidence for these indirect comparisons may lead to bias if any studies are missing for reasons associated with their results. Therefore, for the indirect estimates, we need to account for this potential source of bias, which is represented by the final judgement of the overall bias for pairwise comparisons *observed for other outcomes* or completely *unobserved* in the Pairwise Comparisons Table. We copy the final judgements from column 5 of the Pairwise Comparisons Table (see Table 2 for example) into column 4 of the ROB-MEN Table (see Table 3) of our illustrative example, and we consider only those of the indirect estimates. Three estimates were at suspected bias favouring CCTA, CMR and SPECT-MPI.

#### Small-study effects in network meta-analysis

To evaluate small-study effects, we run a network meta-regression model with a measure of precision (e.g. variance or standard error) as the covariate. This model generates an adjusted relative effect by extrapolating the regression line to the smallest observed variance (the ‘largest’ study) independently for each comparison. To assess the presence of small-study effects, we compare the obtained adjusted estimates with the original (unadjusted) estimates by looking at the overlap of their corresponding confidence (or credible) intervals. A lack of overlap between the two intervals (or between one estimate and the interval for the other estimate) is an indication that effect estimates differ between smaller and larger studies. Note that this approach assumes there is no other explanation for the difference between the original, and the adjusted estimates, i.e. other covariates do not explain it. The evaluation of small-study effects is reported in the penultimate column of the ROB-MEN Table (Table 3), with levels indicating whether there is evidence of small-study effects and, if so, which treatment is favoured by the small studies.

For the example of non-invasive diagnostic modalities, we ran a network meta-regression model using the variance of the estimate (pooled variance for multi-arm studies) as a covariate to investigate small-study effects in the whole network. The adjusted estimates via extrapolation to the smallest observed variance are reported in column 6 of the ROB-MEN Table next to the original network meta-analysis summary effect (column 5 in Table 3). None of the network meta-regression estimates are markedly different from their unadjusted counterparts, and the credible intervals for estimates overlap. Therefore, “No evidence of small-study effects” is reported in column 7 for all the estimates.

#### Overall risk of bias for network meta-analysis estimates

We propose rules for assigning a final judgement on the overall risk of bias due to missing evidence for estimates which are described in Table 5. If there is a substantial contribution from evidence with suspected bias (column 3), we have concerns regarding the risk of bias for that estimate. Suppose this contribution is split between evidence with bias favouring one of the treatments and evidence with bias favouring the other treatment. In that case, the biases may cancel out, assuming the bias is about the same in the two directions. Concerns about the risk of bias are then defined by the overall bias of unobserved comparisons in column 4 (for indirect estimates) and the evidence about small-study effects (column 7). The final judgements for the overall risk of bias are reported in column 8 (see Table 3). The reviewer can decide to follow our proposed rules to assign the overall risk of bias level but, if “stricter” or “more relaxed” approaches are preferred, they can also reach their final judgement based on their own reasoning. Whatever their reasoning, every choice and assessment should be justified and clearly described.

**Table 5:**
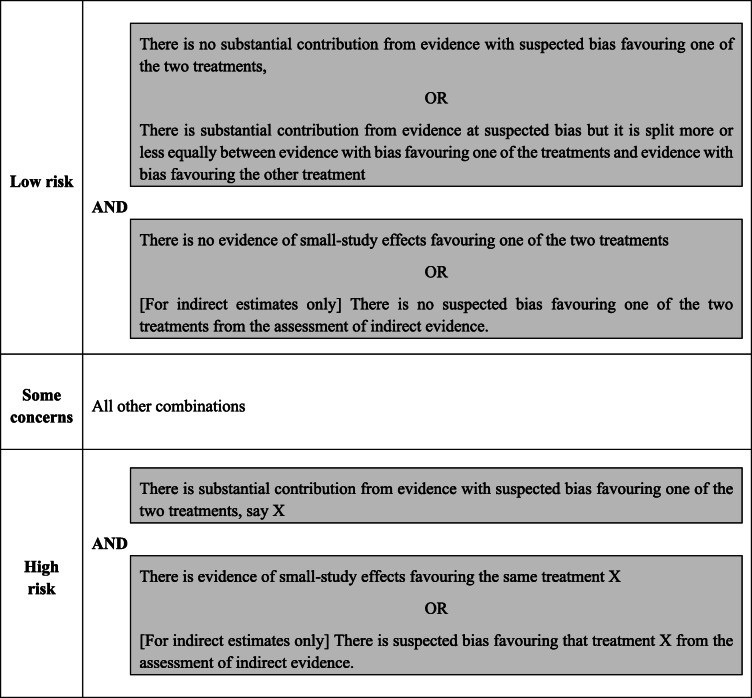
Proposed rules for judging the overall risk of bias due to missing evidence for network meta-analysis estimates

In the example of non-invasive diagnostic modalities most of the mixed estimates have substantial contributions from biased evidence favouring one of the two treatments. Still, there was no evidence of small-study effects for any of the estimates, so we have some concerns about the risk of bias due to missing evidence. The exceptions are exercise ECG vs standard care, Exercise ECG vs stress echo and SPECT-MPI vs standard care. There, the level was decreased to “Low risk” due to lack of substantial contribution from biased evidence favouring either one of the two treatments. Similarly, we assign “Some concerns” to indirect estimates, where a substantial contribution from biased evidence was favouring one of the two treatments. For CMR vs stress echo, the level was increased to “High risk” because of the additional bias from the corresponding indirect comparison assessed in the Pairwise Comparisons Table (Table 2), despite the fact that there is no evidence of small-study effects. The other indirect estimates were assigned a level of “Low risk” of bias because (i) there was no substantial contribution from biased evidence or it cancelled each other out, (ii) there was no additional bias from the indirect comparison assessed in the Pairwise Comparisons Table (Table 2), and (iii) there was no evidence of small-study effects. The final judgements on the overall risk of bias due to missing evidence are reported in column 8 of Table 3.

## Results

### Application of ROB-MEN to a network of antidepressants

We applied the ROB-MEN tool to a network of head-to-head studies (i.e. trials of active interventions) of 18 antidepressants [17]. The outcome of interest is the response to treatment defined as a reduction of at least 50% in the score between baseline and week 8 on a standardised rating scale for depression [30].

#### Pairwise comparisons table

There are 153 possible comparisons between the 18 drugs. Seventy compared the response to the antidepressant (group A) and 2 (amitriptyline vs bupropion and amitriptyline vs nefazodone, group B) compared other outcomes (dropouts and remission). The remaining 82 possible comparisons were not covered in any of the studies (“unobserved”, group C) (see Additional file 7).

We carried out the within-study assessment of bias due to missing evidence for the two comparisons in the “observed for other outcomes” group (*no bias detected*) and for the comparisons in the group “observed for this outcome” for which extra studies were identified that did not report the outcome of interest. We judged four of these to be potentially biased because the extra studies did not report the full results and were sponsored by the company manufacturing the drug favoured by the bias. We judged the other four comparisons as *no bias detected*: the unavailable results were unlikely to be missing due to non-significant *p*-values or the directions of the results and unlikely to affect the overall results. For example, selective outcome reporting bias was suspected for an additional study of fluoxetine versus paroxetine but unlikely to affect the synthesised results given its small sample size (21 participants) relative to the total sample size (1364 participants). We assigned all other comparisons observed for this outcome a level of *no bias detected* in this step. The within-study assessment of bias was not applicable to the 82 unobserved comparisons.

The across-study assessment of bias was carried out for all comparisons. We considered that bias, when suspected, would favour the newest drug, following the novel agent bias principle. The exceptions were comparisons where agomelatine, paroxetine, bupropion, and vortioxetine were the newest drug because the authors obtained all unpublished data from the manufacturers. This qualitative consideration took priority over findings from contour-enhanced funnel plots and tests for small-study effects for comparisons with at least 10 studies. Based on the findings from these statistical techniques, neither amitriptyline versus fluoxetine nor citalopram versus escitalopram would be judged at suspected bias. We nevertheless agreed our judgement from the across-study assessment of bias for both comparisons as *suspected bias favouring the newest drug* because the review authors could not exclude the possibility of hidden studies with unfavourable results towards the newer drug in the comparison (fluoxetine and escitalopram).

Considering the previous assessments, most of the pairwise comparisons were considered at *suspected bias favouring the newest drug*. The only ones judged with *no bias detected* were all comparisons involving agomelatine and vortioxetine, as well as other 12 comparisons involving other drugs. The judgements for all pairwise comparisons are reported in the last column of the Pairwise Comparisons Table (Additional file 7).

#### ROB-MEN Table

Once the Pairwise Comparison Table is complete with all judgements, we integrate them in the ROB-MEN Table. First, the overall risk of bias judgements for comparisons with direct evidence are combined with the results from the contribution matrix to calculate for each network meta-analysis estimate the contribution coming from direct evidence at suspected bias favouring either of the two treatments and in total. We considered an estimate to have substantial contribution from evidence at suspected bias favouring one of the two treatments in the contrast if the difference between the first and second column (contribution from evidence at suspected bias favouring first and favouring second treatment, respectively) was at least 15 percentage points.

The bias assessment for indirect evidence is only considered for the “only indirect” estimates and is copied from the last column of the Pairwise Comparison Table. This potential risk for “missing studies” is particularly important for the indirect estimates because it drives the bias evaluation to a “high risk” level in case there is also substantial contribution from direct evidence with suspected bias in the same direction.

The last part of the risk of bias assessment for the network estimate involves running a network meta-regression model to evaluate the presence (or absence) of small-study effects. We run the model using the smallest observed variance as a covariate and assuming unrelated coefficients. All estimates and their adjusted counterpart were similar, and their credible intervals had a good level of overlap, providing no evidence of small-study effects.

Following the rules set out in Table 4, we assign the final judgements on the overall risk of bias due to missing evidence to the estimates and report it in the last column of the ROB-MEN Table (Additional file 8). Overall, the risk of bias for most estimates was classified as *some concerns* or *low risk*. In particular, none of the comparisons involving agomelatine, paroxetine, venlafaxine, or vortioxetine were at high risk of bias. All 153 network meta-analysis estimates with their relative ROB-MEN levels are reported in Table 6.

**Table 6:**
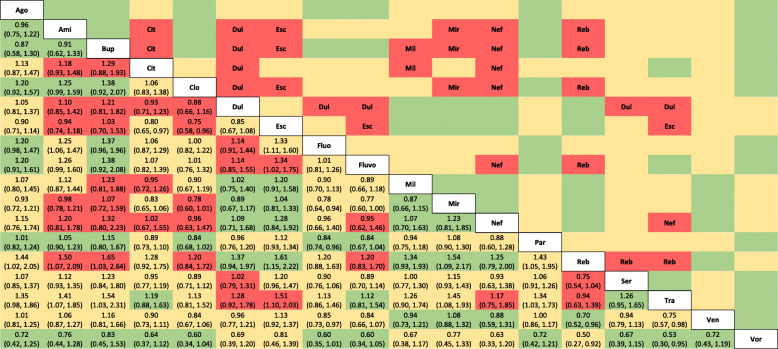
League table of the network estimates and corresponding risk of bias due to missing evidence for the network of 18 antidepressants

The values in the lower triangle represent the relative treatment effect (odds ratios and 95% credible intervals) of the treatment on the top (column) versus the treatment on the row. Colours indicate the ROB-MEN levels: green = low risk; yellow: some concerns; red = high risk. Names in the upper triangle indicate the treatment favoured by the bias in the high risk estimates (red cells). Risk of bias assessments were obtained using the Shiny app. Ago, agomelatine; Ami, amitriptyline; Bup, bupropion; Cit, citalopram; Clo, clomipramine; Dul, duloxetine; Esc, escitalopram; Fluo, fluoxetine; Fluvo, fluvoxamine; Mil, milnacipran; Mir, mirtazapine; Nef, nefazodone; Par, paroxetine; Reb, reboxetine; Ser, sertraline; Tra, trazodone; Ven, venlafaxine; Vor, vortioxetine

## Discussion

To our knowledge, ROB-MEN is the first tool for assessing the risk of bias due to missing evidence in network meta-analysis. ROB-MEN builds on an approach recently proposed for pairwise meta-analysis [1, 10] and adapts it to the network setting. Specifically, the assessments for selective outcome reporting and publication bias in pairwise comparisons are combined with (i) the percentage contribution of direct evidence for each pairwise comparison to the network meta-analysis estimates, (ii) evidence about the presence of small-study effects, and (iii) any bias arising from unobserved comparisons.

Our examples demonstrate that the tool applies to different network meta-analyses, including very large and complex networks, for which assessing the risk of bias can be lengthy and labour-intensive. We developed an R Shiny web application [24] to facilitate the ROB-MEN use. Once the user has evaluated the risk of bias for all pairwise comparisons and estimates, the app produces the Pairwise Comparisons and ROB-MEN Table. The ROB-MEN tool is also incorporated in the reporting bias domain of the CINeMA framework and software [18, 19].

ROB-MEN is not applicable in situations where an intervention of interest is disconnected from the network. It was not designed to cover comparisons involving disconnected interventions. In case of disconnected networks, we recommend to evaluate each subnetwork separately. Like for any other evaluation of results’ credibility in evidence synthesis, many of the judgements in the ROB-MEN process involve subjective decisions. Judging bias due to missing evidence is challenging, particularly for publication bias, as reviewers will often not know about unpublished studies. However, the subjectivity of our approach, specifically in the pairwise comparisons step, is shared by other approaches, as described in the Cochrane Handbook and ROB-ME tool [1, 10]. Also, the novel quantitative methods, the contribution matrix [23] and network meta-regression that we integrated into the assessment rely less on the reviewer's subjectivity.

## Conclusions

We encourage the evidence-synthesis community to conduct studies of the reliability and reproducibility of the ROB-MEN tool. We recommend reviewers specify the criteria used and explain the reasoning behind the judgements to enhance transparency. We believe that ROB-MEN will help those performing network meta-analyses reach better-informed conclusions and enhance the toolbox of available methods for evaluating the credibility of network meta-analysis results.

## Supplementary Information

**Additional file 1.** Network graph, methods and forest plot for the network meta-analysis of non-invasive diagnostic modalities for the detection of coronary artery disease in patients with low risk acute coronary syndromes.

**Additional file 2.** Instructions for filling in the Pairwise Comparisons Table.

**Additional file 3.** Instructions for filling in the ROB-MEN Table.

**Additional file 4.** Description of the judgements from the across-study assessment of bias for the example of non-invasive diagnostic modalities for detection of coronary artery disease in patients with low risk acute coronary syndromes.

**Additional file 5.** Flow chart for assessing overall risk of bias due to missing evidence in pairwise comparisons.

**Additional file 6.** Contribution matrix for the network of non-invasive diagnostic modalities for coronary artery disease in patients with low risk acute coronary syndrome.

**Additional file 7.** Pairwise Comparisons Table for the network of 18 antidepressants.

**Additional file 8.** ROB-MEN Table for the network of 18 antidepressants.

## Data Availability

Data sharing not applicable as no new datasets were generated for this study.

## Abbreviations

ROB-ME: Risk Of Bias due to Missing Evidence
ROB-MEN: Risk Of Bias due to Missing Evidence in Network meta-analysis
CINeMA: Confidence In Network Meta-Analysis
ECG: Electrocardiogram
SPECT-MPI: Single-photon emission computed tomography-myocardial perfusion imaging
CCTA: Coronary computed tomographic angiography
CMR: Cardiovascular magnetic resonance
Stress echo: Stress echocardiography
