# Supplementary material for: ROB-MEN: a tool to assess risk of bias due to missing evidence in network meta-analysis
Source: BMC Med. 2021 Nov 23;19:304. doi: 10.1186/s12916-021-02166-3 (PMC8609747; doi:10.1186/s12916-021-02166-3)
Supplement: Supplementary file 1 — Additional file 1. Network graph, methods and forest plot for the network meta-analysis of non-invasive diagnostic modalities for the detection of coronary artery disease in patients with low risk acute coronary syndromes. [file 12916_2021_2166_MOESM1_ESM.docx]

Network graph, methods and forest plot for the network meta-analysis of non-invasive diagnostic modalities for the detection of coronary artery disease in patients with low risk acute coronary syndromes. ECG: electrocardiogram; SPECT-MPI: single photon emission computed tomography-myocardial perfusion imaging; CCTA: coronary computed tomographic angiography; CMR: cardiovascular magnetic resonance; Echo: echocardiography.

The network was reanalysed by fitting Bayesian random-effects models for network meta-analysis using the BUGSnet package in R. Summary odds ratios (ORs) and 95% credible intervals (CI) were estimated from binomial likelihoods models with common heterogeneity using an independent normal prior distribution with mean 0 and standard deviation 15*u* for the treatment effect and a uniform distribution with range 0 to *u* for the heterogeneity, where *u* represents the largest maximum likelihood estimator in single trials, as recommended by van Valkenhoef et al (*Res. Syn. Meth.* 2012, 3(4):285-99). The adjusted OR are estimated from a network meta-regression model using the smallest observed variance as a covariate and assuming unrelated coefficients. The prespecified prior for the unrelated regression coefficients is a $t\left( 0, u^{2}, 1 \right)$ where *u* is again the largest maximum likelihood estimator in single trials. As reported in the original publication by Siontis et al. (*BMJ* 2018, 360:k504), there was no evidence of major inconsistency.

*Comparisons with direct evidence*: CCTA vs exercise ECG, CCTA vs SPECT-MPI, CCTA vs standard care, CMR vs standard care, exercise ECG vs standard care, exercise ECG vs stress echo, SPECT-MPI vs standard care, standard care vs stress echo.

*Comparisons with indirect evidence*: CCTA vs CMR, CCTA vs stress echo, CMR vs exercise ECG, CMR vs SPECT-MPI, CMR vs stress echo, exercise ECG vs SPECT-MPI, SPECT-MPI vs stress echo.
