# Supplementary material for: ROB-MEN: a tool to assess risk of bias due to missing evidence in network meta-analysis
Source: BMC Med. 2021 Nov 23;19:304. doi: 10.1186/s12916-021-02166-3 (PMC8609747; doi:10.1186/s12916-021-02166-3)
Supplement: Supplementary file 2 — Additional file 2. Instructions for filling in the Pairwise Comparisons Table. [file 12916_2021_2166_MOESM2_ESM.docx]

Instructions for filling in the Pairwise Comparisons Table

| Task | Implementation in R Shiny web application |
| --- | --- |
| List all possible pairwise comparisons between the interventions involved in the network and organize them in three groups “*observed for this outcome”, “observed for other outcomes”, “unobserved*”. | Automated |
| Enter in column 1 the number of studies (and total number of participants randomized in brackets) reporting the outcome of interest for the comparison. In column 2 enter the total number of studies identified for the comparison (and the relevant total number of participants randomized in brackets). Enter 0 in column 1 for comparisons “observed for other outcomes” and “unobserved” and in column 2 for “unobserved” comparisons. | Automated |
| Assess the level of risk for the within-study assessment of bias (selective outcome reporting) using a classification system and enter in column 3 “NA”, “no bias detected”, or “suspected bias favouring treatment X” according to which treatment is favoured by the bias. | Manual/  automated |
| Assess the level of bias due to across-study assessment of bias (publication bias) and enter in column 4 “no bias detected” or “suspected bias favouring treatment X” according to which treatment is favoured by the bias. | Manual |
| Merge the assessments for the within-study and the across-study assessment of bias, as applicable, following the algorithm in Additional file 5. For each comparison, enter in column 5 either “no bias detected” or “suspected bias favouring treatment X”. | Automated/ manual |
