# Supplementary material for: ROB-MEN: a tool to assess risk of bias due to missing evidence in network meta-analysis
Source: BMC Med. 2021 Nov 23;19:304. doi: 10.1186/s12916-021-02166-3 (PMC8609747; doi:10.1186/s12916-021-02166-3)
Supplement: Supplementary file 3 — Additional file 3. Instructions for filling in the ROB-MEN Table. [file 12916_2021_2166_MOESM3_ESM.docx]

Instructions for filling in the ROB-MEN Table

| Task | Implementation in R Shiny web application |
| --- | --- |
| List all network estimates and organize them into two groups, “*mixed/only direct*” and *“only indirect”*. | Automated |
| Enter in column 1 the percentage contribution of direct evidence with suspected bias favouring the first treatment, and in column 2 the percentage contribution of direct evidence favouring the second treatment. | Automated |
| Evaluate the contribution from comparisons with a suspected bias to each estimate and enter in column 3 *“No substantial contribution from bias”, “Substantial contribution from bias balanced”,* or *“Substantial contribution from bias favouring X”* according to the treatment favoured*.* | Manual |
| Copy the final judgements (“no bias detected” or “suspected bias favouring treatment X” according to the treatment favoured) from column 6 of the Pairwise Comparisons Table to column 4 of the ROB-MEN Table only for comparisons with indirect evidence. | Automated |
| Run a network meta-regression model for small-study effects and enter the NMA estimates adjusted for the most precise study in column 6, alongside the relative NMA summary effect in column 5. | Automated |
| Evaluate the presence or absence of small-study effects and enter in column 7 “No evidence of small-study effects” or “Small-study effects favouring treatment X” according to the treatment favoured by the small studies. | Manual |
| For each NMA estimate, enter in column 8 “high risk”, “some concerns”, or “low risk” according to the algorithm rules in Table 4 of the main text. | Automated |
