# Supplementary material for: ROB-MEN: a tool to assess risk of bias due to missing evidence in network meta-analysis
Source: BMC Med. 2021 Nov 23;19:304. doi: 10.1186/s12916-021-02166-3 (PMC8609747; doi:10.1186/s12916-021-02166-3)
Supplement: Supplementary file 4 — Additional file 4. Description of the judgements from the across-study assessment of bias for the example of non-invasive diagnostic modalities for detection of coronary artery disease in patients with low risk acute coronary syndromes. [file 12916_2021_2166_MOESM4_ESM.docx]

**Description of the judgements from the across-study assessment of bias for the example of non-invasive diagnostic modalities for detection of coronary artery disease in patients with low risk acute coronary syndromes.** Abbreviations: ECG: electrocardiogram; CCTA: coronary computed tomographic angiography; CMR: cardiovascular magnetic resonance; SPECT-MPI: single-photon emission computed tomography-myocardial perfusion imaging; Stress Echo: stress echocardiography.

In this example, we considered CCTA vs SPECT-MPI, CCTA vs standard care, and CCTA vs stress echo to be at *suspected bias favouring* CCTA because the latter is a new non-invasive, easily accessible imaging modality. We presumed that any study involving this diagnostic intervention reporting unfavourable results for the CCTA would be at considerable risk of remaining unpublished.

We also considered CMR vs standard care and CMR vs stress echo to be at *suspected bias favouring* CMR for similar reasons as above. Standard care vs stress echo may be biased in favour of stress echo as this was the current state-of-the-art method with higher diagnostic accuracy at the time of the corresponding trials. Besides, the standard of care can refer to very different strategies as it is based on the discretion of the study clinicians or locally applied diagnostic strategies, so potential bias introduced by this “mixed” intervention cannot be excluded.

Finally, we judged exercise ECG vs SPECT-MPI to be at *suspected bias favouring* SPECT-MPI because this was the first widely available non-invasive imaging technology for functional assessment of the heart and was considered the gold-standard method for several years without any strong evidence of clinical benefit over other methods.

We assigned *no bias detected* to all other comparisons. We considered it unlikely that for these comparisons, the studies would remain unpublished if carried out.
