# Supplementary material for: ROB-MEN: a tool to assess risk of bias due to missing evidence in network meta-analysis
Source: BMC Med. 2021 Nov 23;19:304. doi: 10.1186/s12916-021-02166-3 (PMC8609747; doi:10.1186/s12916-021-02166-3)
Supplement: Supplementary file 5 — Additional file 5. Flow chart for assessing overall risk of bias due to missing evidence in pairwise comparisons. [file 12916_2021_2166_MOESM5_ESM.docx]

Flow chart for assessing overall risk of bias due to missing evidence in pairwise comparisons

**No bias detected / NA**

**Suspected bias favouring X**

**No bias detected**

**Suspected bias favouring X**

**No bias detected**

**Suspected bias favouring X**
