# Supplementary material for: ROB-MEN: a tool to assess risk of bias due to missing evidence in network meta-analysis
Source: BMC Med. 2021 Nov 23;19:304. doi: 10.1186/s12916-021-02166-3 (PMC8609747; doi:10.1186/s12916-021-02166-3)
Supplement: Supplementary file 6 — Additional file 6. Contribution matrix for the network of non-invasive diagnostic modalities for coronary artery disease in patients with low risk acute coronary syndrome. [file 12916_2021_2166_MOESM6_ESM.docx]

**Contribution matrix for the network of non-invasive diagnostic modalities for coronary artery disease in patients with low risk acute coronary syndrome.** Cell values are the percentage contribution of the direct comparisons (columns) to the network estimates (rows).

|  | **CCTA vs Exercise ECG** | **CCTA vs**  **SPECT MPI** | **CCTA vs Standard care** | **CMR vs Standard care** | **Exercise ECG vs Standard care** | **Exercise ECG vs Stress Echo** | **SPECT MPI vs Standard care** | **Standard care vs Stress Echo** |
| --- | --- | --- | --- | --- | --- | --- | --- | --- |
| **CCTA vs CMR** | 0.81 | 5.41 | 40.52 | 46.75 | 0.53 | 0.28 | 5.41 | 0.28 |
| **CCTA vs Exercise ECG** | 51.64 | 2.02 | 18.16 | 0 | 14.2 | 5.98 | 2.02 | 5.98 |
| **CCTA vs SPECT-MPI** | 0.64 | 34.05 | 31.9 | 0 | 0.42 | 0.22 | 32.55 | 0.22 |
| **CCTA vs Standard care** | 1.17 | 8.12 | 81.04 | 0 | 0.8 | 0.37 | 8.12 | 0.37 |
| **CCTA vs Stress Echo** | 24.04 | 2.17 | 18.7 | 0 | 8.02 | 32.06 | 2.17 | 12.85 |
| **CMR vs Exercise ECG** | 15.63 | 2.04 | 13.58 | 37.65 | 15 | 7.03 | 2.04 | 7.03 |
| **CMR vs SPECT-MPI** | 0.13 | 5.88 | 5.74 | 46.97 | 0.08 | 0.05 | 41.09 | 0.05 |
| **CMR vs Standard care** | 0 | 0 | 0 | 100 | 0 | 0 | 0 | 0 |
| **CMR vs Stress Echo** | 10.96 | 1.51 | 9.45 | 33.64 | 9.27 | 20.23 | 1.51 | 13.41 |
| **Exercise ECG vs SPECT-MPI** | 20.83 | 12.99 | 7.84 | 0 | 14.83 | 6.95 | 29.62 | 6.95 |
| **Exercise ECG vs Standard care** | 23.1 | 2.72 | 20.38 | 0 | 29.99 | 10.54 | 2.72 | 10.54 |
| **Exercise ECG vs Stress Echo** | 1.14 | 0.15 | 0.99 | 0 | 1.09 | 94.26 | 0.15 | 2.23 |
| **SPECT-MPI vs Standard care** | 0.17 | 8.79 | 8.62 | 0 | 0.11 | 0.06 | 82.19 | 0.06 |
| **SPECT-MPI vs Stress Echo** | 13.6 | 8.46 | 5.14 | 0 | 9.16 | 22.76 | 27.59 | 13.29 |
| **Standard care vs Stress Echo** | 14.49 | 1.89 | 12.6 | 0 | 13.91 | 28.4 | 1.89 | 26.82 |
