# Supplementary material for: ROB-MEN: a tool to assess risk of bias due to missing evidence in network meta-analysis
Source: BMC Med. 2021 Nov 23;19:304. doi: 10.1186/s12916-021-02166-3 (PMC8609747; doi:10.1186/s12916-021-02166-3)
Supplement: Supplementary file 7 — Additional file 7. Pairwise Comparisons Table for the network of 18 antidepressants. [file 12916_2021_2166_MOESM7_ESM.docx]

**Pairwise Comparisons Table for the network of 18 antidepressants.** There are 153 possible comparisons between the 18 drugs.

|  | **No. of studies in each comparison** | | **Within-study assessment of bias** | **Across-study assessment of bias** | **Overall bias** |
| --- | --- | --- | --- | --- | --- |
| **Pairwise comparison** | Reporting this outcome (sample size) | Total identified in the SR (total sample size) | Evaluation of selective reporting within studies using signalling questions | Qualitative and quantitative assessment of publication bias | Overall judgement |
| **Group A:  observed for this outcome** | | | | | |
| **Agomelatine Vs. Duloxetine** | 1 ( 418 ) | 1 ( 418 ) | No bias detected | No bias detected | No bias detected |
| **Agomelatine Vs. Escitalopram** | 2 ( 462 ) | 2 ( 462 ) | No bias detected | No bias detected | No bias detected |
| **Agomelatine Vs. Fluoxetine** | 2 ( 1143 ) | 2 ( 1143 ) | No bias detected | No bias detected | No bias detected |
| **Agomelatine Vs. Paroxetine** | 2 ( 747 ) | 2 ( 747 ) | No bias detected | No bias detected | No bias detected |
| **Agomelatine Vs. Venlafaxine** | 2 ( 609 ) | 2 ( 609 ) | No bias detected | No bias detected | No bias detected |
| **Amitriptyline Vs. Fluoxetine** | 12 ( 888 ) | 14 ( 979 ) | Suspected bias favouring Fluoxetine | Suspected bias favouring Fluoxetine | Suspected bias favouring Fluoxetine |
| **Amitriptyline Vs. Fluvoxamine** | 3 ( 337 ) | 3 ( 337 ) | No bias detected | Suspected bias favouring Fluvoxamine | Suspected bias favouring Fluvoxamine |
| **Amitriptyline Vs. Milnacipran** | 2 ( 231 ) | 2 ( 231 ) | No bias detected | Suspected bias favouring Milnacipran | Suspected bias favouring Milnacipran |
| **Amitriptyline Vs. Paroxetine** | 13 ( 1575 ) | 18 ( 1688 ) | No bias detected | No bias detected | No bias detected |
| **Amitriptyline Vs. Sertraline** | 4 ( 709 ) | 4 ( 709 ) | No bias detected | Suspected bias favouring Sertraline | Suspected bias favouring Sertraline |
| **Amitriptyline Vs. Trazodone** | 2 ( 229 ) | 3 ( 269 ) | Suspected bias favouring Trazodone | Suspected bias favouring Trazodone | Suspected bias favouring Trazodone |
| **Amitriptyline Vs. Venlafaxine** | 2 ( 272 ) | 2 ( 272 ) | No bias detected | Suspected bias favouring Venlafaxine | Suspected bias favouring Venlafaxine |
| **Bupropion Vs. Fluoxetine** | 1 ( 123 ) | 1 ( 123 ) | No bias detected | No bias detected | No bias detected |
| **Bupropion Vs. Paroxetine** | 1 ( 140 ) | 2 ( 218 ) | No bias detected | No bias detected | No bias detected |
| **Bupropion Vs. Sertraline** | 1 ( 16 ) | 1 ( 16 ) | No bias detected | Suspected bias favouring Sertraline | Suspected bias favouring Sertraline |
| **Bupropion Vs. Trazodone** | 1 ( 124 ) | 1 ( 124 ) | No bias detected | Suspected bias favouring Trazodone | Suspected bias favouring Trazodone |
| **Bupropion Vs. Venlafaxine** | 1 ( 348 ) | 1 ( 348 ) | No bias detected | Suspected bias favouring Venlafaxine | Suspected bias favouring Venlafaxine |
| **Citalopram Vs. Clomipramine** | 1 ( 114 ) | 1 ( 114 ) | No bias detected | Suspected bias favouring Citalopram | Suspected bias favouring Citalopram |
| **Citalopram Vs. Escitalopram** | 10 ( 1511 ) | 10 ( 1511 ) | No bias detected | Suspected bias favouring Escitalopram | Suspected bias favouring Escitalopram |
| **Citalopram Vs. Fluoxetine** | 2 ( 673 ) | 2 ( 673 ) | No bias detected | Suspected bias favouring Citalopram | Suspected bias favouring Citalopram |
| **Citalopram Vs. Fluvoxamine** | 1 ( 217 ) | 1 ( 217 ) | No bias detected | Suspected bias favouring Citalopram | Suspected bias favouring Citalopram |
| **Citalopram Vs. Mirtazapine** | 1 ( 270 ) | 2 ( 322 ) | Suspected bias favouring Mirtazapine | Suspected bias favouring Mirtazapine | Suspected bias favouring Mirtazapine |
| **Citalopram Vs. Reboxetine** | 1 ( 359 ) | 1 ( 359 ) | No bias detected | Suspected bias favouring Reboxetine | Suspected bias favouring Reboxetine |
| **Citalopram Vs. Sertraline** | 3 ( 596 ) | 3 ( 596 ) | No bias detected | Suspected bias favouring Citalopram | Suspected bias favouring Citalopram |
| **Citalopram Vs. Venlafaxine** | 2 ( 204 ) | 2 ( 204 ) | No bias detected | Suspected bias favouring Venlafaxine | Suspected bias favouring Venlafaxine |
| **Clomipramine Vs. Fluoxetine** | 4 ( 347 ) | 4 ( 347 ) | No bias detected | Suspected bias favouring Fluoxetine | Suspected bias favouring Fluoxetine |
| **Clomipramine Vs. Fluvoxamine** | 2 ( 83 ) | 2 ( 83 ) | No bias detected | Suspected bias favouring Fluvoxamine | Suspected bias favouring Fluvoxamine |
| **Clomipramine Vs. Milnacipran** | 1 ( 107 ) | 1 ( 107 ) | No bias detected | Suspected bias favouring Milnacipran | Suspected bias favouring Milnacipran |
| **Clomipramine Vs. Paroxetine** | 6 ( 1455 ) | 6 ( 1455 ) | No bias detected | No bias detected | No bias detected |
| **Clomipramine Vs. Sertraline** | 2 ( 272 ) | 2 ( 272 ) | No bias detected | Suspected bias favouring Sertraline | Suspected bias favouring Sertraline |
| **Clomipramine Vs. Trazodone** | 1 ( 115 ) | 1 ( 115 ) | No bias detected | Suspected bias favouring Trazodone | Suspected bias favouring Trazodone |
| **Clomipramine Vs. Venlafaxine** | 2 ( 215 ) | 2 ( 215 ) | No bias detected | Suspected bias favouring Venlafaxine | Suspected bias favouring Venlafaxine |
| **Duloxetine Vs. Escitalopram** | 2 ( 573 ) | 2 ( 573 ) | No bias detected | Suspected bias favouring Duloxetine | Suspected bias favouring Duloxetine |
| **Duloxetine Vs. Paroxetine** | 2 ( 759 ) | 2 ( 759 ) | No bias detected | Suspected bias favouring Duloxetine | Suspected bias favouring Duloxetine |
| **Duloxetine Vs. Venlafaxine** | 2 ( 836 ) | 2 ( 836 ) | No bias detected | Suspected bias favouring Duloxetine | Suspected bias favouring Duloxetine |
| **Escitalopram Vs. Fluoxetine** | 2 ( 445 ) | 3 ( 475 ) | Suspected bias favouring Escitalopram | Suspected bias favouring Escitalopram | Suspected bias favouring Escitalopram |
| **Escitalopram Vs. Paroxetine** | 2 ( 784 ) | 2 ( 784 ) | No bias detected | Suspected bias favouring Escitalopram | Suspected bias favouring Escitalopram |
| **Escitalopram Vs. Sertraline** | 2 ( 355 ) | 2 ( 355 ) | No bias detected | Suspected bias favouring Escitalopram | Suspected bias favouring Escitalopram |
| **Escitalopram Vs. Venlafaxine** | 2 ( 495 ) | 2 ( 495 ) | No bias detected | Suspected bias favouring Escitalopram | Suspected bias favouring Escitalopram |
| **Fluoxetine Vs. Fluvoxamine** | 2 ( 284 ) | 2 ( 284 ) | No bias detected | Suspected bias favouring Fluoxetine | Suspected bias favouring Fluoxetine |
| **Fluoxetine Vs. Milnacipran** | 2 ( 490 ) | 2 ( 490 ) | No bias detected | Suspected bias favouring Milnacipran | Suspected bias favouring Milnacipran |
| **Fluoxetine Vs. Mirtazapine** | 5 ( 615 ) | 5 ( 615 ) | No bias detected | Suspected bias favouring Mirtazapine | Suspected bias favouring Mirtazapine |
| **Fluoxetine Vs. Nefazodone** | 3 ( 161 ) | 3 ( 161 ) | No bias detected | Suspected bias favouring Nefazodone | Suspected bias favouring Nefazodone |
| **Fluoxetine Vs. Paroxetine** | 9 ( 1364 ) | 10 ( 1385 ) | No bias detected | No bias detected | No bias detected |
| **Fluoxetine Vs. Reboxetine** | 2 ( 253 ) | 2 ( 253 ) | No bias detected | Suspected bias favouring Reboxetine | Suspected bias favouring Reboxetine |
| **Fluoxetine Vs. Sertraline** | 6 ( 1221 ) | 6 ( 1221 ) | No bias detected | Suspected bias favouring Sertraline | Suspected bias favouring Sertraline |
| **Fluoxetine Vs. Trazodone** | 4 ( 234 ) | 4 ( 234 ) | No bias detected | Suspected bias favouring Trazodone | Suspected bias favouring Trazodone |
| **Fluoxetine Vs. Venlafaxine** | 9 ( 2538 ) | 10 ( 2804 ) | Suspected bias favouring Venlafaxine | Suspected bias favouring Venlafaxine | Suspected bias favouring Venlafaxine |
| **Fluvoxamine Vs. Milnacipran** | 2 ( 239 ) | 2 ( 239 ) | No bias detected | Suspected bias favouring Milnacipran | Suspected bias favouring Milnacipran |
| **Fluvoxamine Vs. Mirtazapine** | 2 ( 412 ) | 2 ( 412 ) | No bias detected | Suspected bias favouring Mirtazapine | Suspected bias favouring Mirtazapine |
| **Fluvoxamine Vs. Paroxetine** | 2 ( 180 ) | 2 ( 180 ) | No bias detected | No bias detected | No bias detected |
| **Fluvoxamine Vs. Sertraline** | 2 ( 185 ) | 2 ( 185 ) | No bias detected | Suspected bias favouring Sertraline | Suspected bias favouring Sertraline |
| **Fluvoxamine Vs. Venlafaxine** | 1 ( 111 ) | 1 ( 111 ) | No bias detected | Suspected bias favouring Venlafaxine | Suspected bias favouring Venlafaxine |
| **Milnacipran Vs. Paroxetine** | 2 ( 1207 ) | 2 ( 1207 ) | No bias detected | Suspected bias favouring Milnacipran | Suspected bias favouring Milnacipran |
| **Milnacipran Vs. Sertraline** | 1 ( 53 ) | 1 ( 53 ) | No bias detected | Suspected bias favouring Milnacipran | Suspected bias favouring Milnacipran |
| **Mirtazapine Vs. Paroxetine** | 5 ( 916 ) | 5 ( 916 ) | No bias detected | Suspected bias favouring Mirtazapine | Suspected bias favouring Mirtazapine |
| **Mirtazapine Vs. Sertraline** | 1 ( 346 ) | 1 ( 346 ) | No bias detected | Suspected bias favouring Mirtazapine | Suspected bias favouring Mirtazapine |
| **Mirtazapine Vs. Trazodone** | 1 ( 200 ) | 1 ( 200 ) | No bias detected | Suspected bias favouring Mirtazapine | Suspected bias favouring Mirtazapine |
| **Mirtazapine Vs. Venlafaxine** | 2 ( 533 ) | 2 ( 533 ) | No bias detected | Suspected bias favouring Venlafaxine | Suspected bias favouring Venlafaxine |
| **Nefazodone Vs. Paroxetine** | 2 ( 246 ) | 2 ( 246 ) | No bias detected | Suspected bias favouring Nefazodone | Suspected bias favouring Nefazodone |
| **Nefazodone Vs. Sertraline** | 1 ( 160 ) | 1 ( 160 ) | No bias detected | Suspected bias favouring Nefazodone | Suspected bias favouring Nefazodone |
| **Paroxetine Vs. Reboxetine** | 1 ( 325 ) | 1 ( 325 ) | No bias detected | Suspected bias favouring Reboxetine | Suspected bias favouring Reboxetine |
| **Paroxetine Vs. Sertraline** | 2 ( 545 ) | 2 ( 545 ) | No bias detected | No bias detected | No bias detected |
| **Paroxetine Vs. Trazodone** | 2 ( 333 ) | 2 ( 333 ) | No bias detected | No bias detected | No bias detected |
| **Paroxetine Vs. Venlafaxine** | 2 ( 475 ) | 2 ( 475 ) | No bias detected | Suspected bias favouring Venlafaxine | Suspected bias favouring Venlafaxine |
| **Reboxetine Vs. Venlafaxine** | 1 ( 167 ) | 1 ( 167 ) | No bias detected | Suspected bias favouring Venlafaxine | Suspected bias favouring Venlafaxine |
| **Sertraline Vs. Trazodone** | 1 ( 122 ) | 1 ( 122 ) | No bias detected | Suspected bias favouring Sertraline | Suspected bias favouring Sertraline |
| **Sertraline Vs. Venlafaxine** | 3 ( 470 ) | 3 ( 470 ) | No bias detected | Suspected bias favouring Venlafaxine | Suspected bias favouring Venlafaxine |
| **Trazodone Vs. Venlafaxine** | 1 ( 112 ) | 1 ( 112 ) | No bias detected | Suspected bias favouring Venlafaxine | Suspected bias favouring Venlafaxine |
| **Venlafaxine Vs. Vortioxetine** | 1 ( 443 ) | 1 ( 443 ) | No bias detected | No bias detected | No bias detected |
| **Group B: observed for other outcomes** | | | | | |
| **Amitriptyline Vs. Bupropion** | 0 ( 0 ) | 1 ( 118 ) | No bias detected | No bias detected | No bias detected |
| **Amitriptyline Vs. Nefazodone** | 0 ( 0 ) | 1 ( 37 ) | No bias detected | Suspected bias favouring Nefazodone | Suspected bias favouring Nefazodone |
| **Group C: Unobserved** | | | | | |
| **Agomelatine Vs. Amitriptyline** | 0 ( 0 ) | 0 ( 0 ) | NA | No bias detected | No bias detected |
| **Agomelatine Vs. Bupropion** | 0 ( 0 ) | 0 ( 0 ) | NA | No bias detected | No bias detected |
| **Agomelatine Vs. Citalopram** | 0 ( 0 ) | 0 ( 0 ) | NA | No bias detected | No bias detected |
| **Agomelatine Vs. Clomipramine** | 0 ( 0 ) | 0 ( 0 ) | NA | No bias detected | No bias detected |
| **Agomelatine Vs. Fluvoxamine** | 0 ( 0 ) | 0 ( 0 ) | NA | No bias detected | No bias detected |
| **Agomelatine Vs. Milnacipran** | 0 ( 0 ) | 0 ( 0 ) | NA | No bias detected | No bias detected |
| **Agomelatine Vs. Mirtazapine** | 0 ( 0 ) | 0 ( 0 ) | NA | No bias detected | No bias detected |
| **Agomelatine Vs. Nefazodone** | 0 ( 0 ) | 0 ( 0 ) | NA | No bias detected | No bias detected |
| **Agomelatine Vs. Reboxetine** | 0 ( 0 ) | 0 ( 0 ) | NA | No bias detected | No bias detected |
| **Agomelatine Vs. Sertraline** | 0 ( 0 ) | 0 ( 0 ) | NA | No bias detected | No bias detected |
| **Agomelatine Vs. Trazodone** | 0 ( 0 ) | 0 ( 0 ) | NA | No bias detected | No bias detected |
| **Agomelatine Vs. Vortioxetine** | 0 ( 0 ) | 0 ( 0 ) | NA | No bias detected | No bias detected |
| **Amitriptyline Vs. Citalopram** | 0 ( 0 ) | 0 ( 0 ) | NA | Suspected bias favouring Citalopram | Suspected bias favouring Citalopram |
| **Amitriptyline Vs. Clomipramine** | 0 ( 0 ) | 0 ( 0 ) | NA | No bias detected | No bias detected |
| **Amitriptyline Vs. Duloxetine** | 0 ( 0 ) | 0 ( 0 ) | NA | Suspected bias favouring Duloxetine | Suspected bias favouring Duloxetine |
| **Amitriptyline Vs. Escitalopram** | 0 ( 0 ) | 0 ( 0 ) | NA | Suspected bias favouring Escitalopram | Suspected bias favouring Escitalopram |
| **Amitriptyline Vs. Mirtazapine** | 0 ( 0 ) | 0 ( 0 ) | NA | Suspected bias favouring Mirtazapine | Suspected bias favouring Mirtazapine |
| **Amitriptyline Vs. Reboxetine** | 0 ( 0 ) | 0 ( 0 ) | NA | Suspected bias favouring Reboxetine | Suspected bias favouring Reboxetine |
| **Amitriptyline Vs. Vortioxetine** | 0 ( 0 ) | 0 ( 0 ) | NA | No bias detected | No bias detected |
| **Bupropion Vs. Citalopram** | 0 ( 0 ) | 0 ( 0 ) | NA | Suspected bias favouring Citalopram | Suspected bias favouring Citalopram |
| **Bupropion Vs. Clomipramine** | 0 ( 0 ) | 0 ( 0 ) | NA | No bias detected | No bias detected |
| **Bupropion Vs. Duloxetine** | 0 ( 0 ) | 0 ( 0 ) | NA | Suspected bias favouring Duloxetine | Suspected bias favouring Duloxetine |
| **Bupropion Vs. Escitalopram** | 0 ( 0 ) | 0 ( 0 ) | NA | Suspected bias favouring Escitalopram | Suspected bias favouring Escitalopram |
| **Bupropion Vs. Fluvoxamine** | 0 ( 0 ) | 0 ( 0 ) | NA | Suspected bias favouring Fluvoxamine | Suspected bias favouring Fluvoxamine |
| **Bupropion Vs. Milnacipran** | 0 ( 0 ) | 0 ( 0 ) | NA | Suspected bias favouring Milnacipran | Suspected bias favouring Milnacipran |
| **Bupropion Vs. Mirtazapine** | 0 ( 0 ) | 0 ( 0 ) | NA | Suspected bias favouring Mirtazapine | Suspected bias favouring Mirtazapine |
| **Bupropion Vs. Nefazodone** | 0 ( 0 ) | 0 ( 0 ) | NA | Suspected bias favouring Nefazodone | Suspected bias favouring Nefazodone |
| **Bupropion Vs. Reboxetine** | 0 ( 0 ) | 0 ( 0 ) | NA | Suspected bias favouring Reboxetine | Suspected bias favouring Reboxetine |
| **Bupropion Vs. Vortioxetine** | 0 ( 0 ) | 0 ( 0 ) | NA | No bias detected | No bias detected |
| **Citalopram Vs. Duloxetine** | 0 ( 0 ) | 0 ( 0 ) | NA | Suspected bias favouring Duloxetine | Suspected bias favouring Duloxetine |
| **Citalopram Vs. Milnacipran** | 0 ( 0 ) | 0 ( 0 ) | NA | Suspected bias favouring Milnacipran | Suspected bias favouring Milnacipran |
| **Citalopram Vs. Nefazodone** | 0 ( 0 ) | 0 ( 0 ) | NA | Suspected bias favouring Nefazodone | Suspected bias favouring Nefazodone |
| **Citalopram Vs. Paroxetine** | 0 ( 0 ) | 0 ( 0 ) | NA | No bias detected | No bias detected |
| **Citalopram Vs. Trazodone** | 0 ( 0 ) | 0 ( 0 ) | NA | Suspected bias favouring Trazodone | Suspected bias favouring Trazodone |
| **Citalopram Vs. Vortioxetine** | 0 ( 0 ) | 0 ( 0 ) | NA | No bias detected | No bias detected |
| **Clomipramine Vs. Duloxetine** | 0 ( 0 ) | 0 ( 0 ) | NA | Suspected bias favouring Duloxetine | Suspected bias favouring Duloxetine |
| **Clomipramine Vs. Escitalopram** | 0 ( 0 ) | 0 ( 0 ) | NA | Suspected bias favouring Escitalopram | Suspected bias favouring Escitalopram |
| **Clomipramine Vs. Mirtazapine** | 0 ( 0 ) | 0 ( 0 ) | NA | Suspected bias favouring Mirtazapine | Suspected bias favouring Mirtazapine |
| **Clomipramine Vs. Nefazodone** | 0 ( 0 ) | 0 ( 0 ) | NA | Suspected bias favouring Nefazodone | Suspected bias favouring Nefazodone |
| **Clomipramine Vs. Reboxetine** | 0 ( 0 ) | 0 ( 0 ) | NA | Suspected bias favouring Reboxetine | Suspected bias favouring Reboxetine |
| **Clomipramine Vs. Vortioxetine** | 0 ( 0 ) | 0 ( 0 ) | NA | No bias detected | No bias detected |
| **Duloxetine Vs. Fluoxetine** | 0 ( 0 ) | 0 ( 0 ) | NA | Suspected bias favouring Duloxetine | Suspected bias favouring Duloxetine |
| **Duloxetine Vs. Fluvoxamine** | 0 ( 0 ) | 0 ( 0 ) | NA | Suspected bias favouring Duloxetine | Suspected bias favouring Duloxetine |
| **Duloxetine Vs. Milnacipran** | 0 ( 0 ) | 0 ( 0 ) | NA | Suspected bias favouring Duloxetine | Suspected bias favouring Duloxetine |
| **Duloxetine Vs. Mirtazapine** | 0 ( 0 ) | 0 ( 0 ) | NA | Suspected bias favouring Duloxetine | Suspected bias favouring Duloxetine |
| **Duloxetine Vs. Nefazodone** | 0 ( 0 ) | 0 ( 0 ) | NA | Suspected bias favouring Duloxetine | Suspected bias favouring Duloxetine |
| **Duloxetine Vs. Reboxetine** | 0 ( 0 ) | 0 ( 0 ) | NA | Suspected bias favouring Duloxetine | Suspected bias favouring Duloxetine |
| **Duloxetine Vs. Sertraline** | 0 ( 0 ) | 0 ( 0 ) | NA | Suspected bias favouring Duloxetine | Suspected bias favouring Duloxetine |
| **Duloxetine Vs. Trazodone** | 0 ( 0 ) | 0 ( 0 ) | NA | Suspected bias favouring Duloxetine | Suspected bias favouring Duloxetine |
| **Duloxetine Vs. Vortioxetine** | 0 ( 0 ) | 0 ( 0 ) | NA | No bias detected | No bias detected |
| **Escitalopram Vs. Fluvoxamine** | 0 ( 0 ) | 0 ( 0 ) | NA | Suspected bias favouring Escitalopram | Suspected bias favouring Escitalopram |
| **Escitalopram Vs. Milnacipran** | 0 ( 0 ) | 0 ( 0 ) | NA | Suspected bias favouring Escitalopram | Suspected bias favouring Escitalopram |
| **Escitalopram Vs. Mirtazapine** | 0 ( 0 ) | 0 ( 0 ) | NA | Suspected bias favouring Escitalopram | Suspected bias favouring Escitalopram |
| **Escitalopram Vs. Nefazodone** | 0 ( 0 ) | 0 ( 0 ) | NA | Suspected bias favouring Escitalopram | Suspected bias favouring Escitalopram |
| **Escitalopram Vs. Reboxetine** | 0 ( 0 ) | 0 ( 0 ) | NA | Suspected bias favouring Escitalopram | Suspected bias favouring Escitalopram |
| **Escitalopram Vs. Trazodone** | 0 ( 0 ) | 0 ( 0 ) | NA | Suspected bias favouring Escitalopram | Suspected bias favouring Escitalopram |
| **Escitalopram Vs. Vortioxetine** | 0 ( 0 ) | 0 ( 0 ) | NA | No bias detected | No bias detected |
| **Fluoxetine Vs. Vortioxetine** | 0 ( 0 ) | 0 ( 0 ) | NA | No bias detected | No bias detected |
| **Fluvoxamine Vs. Nefazodone** | 0 ( 0 ) | 0 ( 0 ) | NA | Suspected bias favouring Nefazodone | Suspected bias favouring Nefazodone |
| **Fluvoxamine Vs. Reboxetine** | 0 ( 0 ) | 0 ( 0 ) | NA | Suspected bias favouring Reboxetine | Suspected bias favouring Reboxetine |
| **Fluvoxamine Vs. Trazodone** | 0 ( 0 ) | 0 ( 0 ) | NA | Suspected bias favouring Fluvoxamine | Suspected bias favouring Fluvoxamine |
| **Fluvoxamine Vs. Vortioxetine** | 0 ( 0 ) | 0 ( 0 ) | NA | No bias detected | No bias detected |
| **Milnacipran Vs. Mirtazapine** | 0 ( 0 ) | 0 ( 0 ) | NA | Suspected bias favouring Milnacipran | Suspected bias favouring Milnacipran |
| **Milnacipran Vs. Nefazodone** | 0 ( 0 ) | 0 ( 0 ) | NA | Suspected bias favouring Milnacipran | Suspected bias favouring Milnacipran |
| **Milnacipran Vs. Reboxetine** | 0 ( 0 ) | 0 ( 0 ) | NA | Suspected bias favouring Reboxetine | Suspected bias favouring Reboxetine |
| **Milnacipran Vs. Trazodone** | 0 ( 0 ) | 0 ( 0 ) | NA | Suspected bias favouring Trazodone | Suspected bias favouring Trazodone |
| **Milnacipran Vs. Venlafaxine** | 0 ( 0 ) | 0 ( 0 ) | NA | Suspected bias favouring Venlafaxine | Suspected bias favouring Venlafaxine |
| **Milnacipran Vs. Vortioxetine** | 0 ( 0 ) | 0 ( 0 ) | NA | No bias detected | No bias detected |
| **Mirtazapine Vs. Nefazodone** | 0 ( 0 ) | 0 ( 0 ) | NA | Suspected bias favouring Nefazodone | Suspected bias favouring Nefazodone |
| **Mirtazapine Vs. Reboxetine** | 0 ( 0 ) | 0 ( 0 ) | NA | Suspected bias favouring Reboxetine | Suspected bias favouring Reboxetine |
| **Mirtazapine Vs. Vortioxetine** | 0 ( 0 ) | 0 ( 0 ) | NA | No bias detected | No bias detected |
| **Nefazodone Vs. Reboxetine** | 0 ( 0 ) | 0 ( 0 ) | NA | Suspected bias favouring Reboxetine | Suspected bias favouring Reboxetine |
| **Nefazodone Vs. Trazodone** | 0 ( 0 ) | 0 ( 0 ) | NA | Suspected bias favouring Nefazodone | Suspected bias favouring Nefazodone |
| **Nefazodone Vs. Venlafaxine** | 0 ( 0 ) | 0 ( 0 ) | NA | Suspected bias favouring Venlafaxine | Suspected bias favouring Venlafaxine |
| **Nefazodone Vs. Vortioxetine** | 0 ( 0 ) | 0 ( 0 ) | NA | No bias detected | No bias detected |
| **Paroxetine Vs. Vortioxetine** | 0 ( 0 ) | 0 ( 0 ) | NA | No bias detected | No bias detected |
| **Reboxetine Vs. Sertraline** | 0 ( 0 ) | 0 ( 0 ) | NA | Suspected bias favouring Reboxetine | Suspected bias favouring Reboxetine |
| **Reboxetine Vs. Trazodone** | 0 ( 0 ) | 0 ( 0 ) | NA | Suspected bias favouring Reboxetine | Suspected bias favouring Reboxetine |
| **Reboxetine Vs. Vortioxetine** | 0 ( 0 ) | 0 ( 0 ) | NA | No bias detected | No bias detected |
| **Sertraline Vs. Vortioxetine** | 0 ( 0 ) | 0 ( 0 ) | NA | No bias detected | No bias detected |
| **Trazodone Vs. Vortioxetine** | 0 ( 0 ) | 0 ( 0 ) | NA | No bias detected | No bias detected |
