# Supplementary material for: ROB-MEN: a tool to assess risk of bias due to missing evidence in network meta-analysis
Source: BMC Med. 2021 Nov 23;19:304. doi: 10.1186/s12916-021-02166-3 (PMC8609747; doi:10.1186/s12916-021-02166-3)
Supplement: Supplementary file 8 — Additional file 8. ROB-MEN Table for the network of 18 antidepressants. [file 12916_2021_2166_MOESM8_ESM.docx]

**ROB-MEN Table for the network of 18 antidepressants**

|  | **1** | **2** | **3** | **4** | **5** | **6** | **7** | **8** |
| --- | --- | --- | --- | --- | --- | --- | --- | --- |
|  | **% contribution of evidence from pairwise comparisons with suspected bias** | | **Evaluation of contribution from evidence with suspected bias** | **Bias assessment for indirect evidence** | **NMA treatment effect** | **NMR treatment effect at the smallest observed variance** | **Evaluation of small-study effects** | **Overall risk of bias** |
| **NMA estimate** | **Favouring first treatment** | **Favouring second treatment** |  |  |  |  |  |  |
| **Mixed/ only direct** | | | | | | | | |
| **Agomelatine Vs. Duloxetine** | 0.00 | 32.38 | Substantial contribution from bias favouring Duloxetine |  | 1.05 (0.81, 1.37) | 1.03 (0.75, 1.37) | No evidence of small-study effects | Some concerns |
| **Agomelatine Vs. Escitalopram** | 0.00 | 25.36 | Substantial contribution from bias favouring Escitalopram |  | 0.9 (0.71, 1.14) | 0.89 (0.67, 1.17) | No evidence of small-study effects | Some concerns |
| **Agomelatine Vs. Fluoxetine** | 0.00 | 3.37 | No substantial contribution from bias |  | 1.2 (0.98, 1.47) | 1.17 (0.94, 1.46) | No evidence of small-study effects | Low risk |
| **Agomelatine Vs. Paroxetine** | 0.00 | 0.00 | No substantial contribution from bias |  | 1.01 (0.82, 1.24) | 1.01 (0.79, 1.27) | No evidence of small-study effects | Low risk |
| **Agomelatine Vs. Venlafaxine** | 0.00 | 22.37 | Substantial contribution from bias favouring Venlafaxine |  | 1.01 (0.81, 1.25) | 1 (0.78, 1.27) | No evidence of small-study effects | Some concerns |
| **Amitriptyline Vs. Fluoxetine** | 0.00 | 39.66 | Substantial contribution from bias favouring Fluoxetine |  | 1.25 (1.06, 1.47) | 1.29 (1.05, 1.58) | No evidence of small-study effects | Some concerns |
| **Amitriptyline Vs. Fluvoxamine** | 0.00 | 20.51 | Substantial contribution from bias favouring Fluvoxamine |  | 1.26 (0.99, 1.6) | 1.31 (0.96, 1.79) | No evidence of small-study effects | Some concerns |
| **Amitriptyline Vs. Milnacipran** | 0.00 | 55.18 | Substantial contribution from bias favouring Milnacipran |  | 1.12 (0.87, 1.44) | 1.17 (0.88, 1.55) | No evidence of small-study effects | Some concerns |
| **Amitriptyline Vs. Paroxetine** | 0.00 | 0.00 | No substantial contribution from bias |  | 1.05 (0.9, 1.23) | 1.11 (0.92, 1.33) | No evidence of small-study effects | Low risk |
| **Amitriptyline Vs. Sertraline** | 0.00 | 37.56 | Substantial contribution from bias favouring Sertraline |  | 1.12 (0.93, 1.35) | 1.12 (0.9, 1.4) | No evidence of small-study effects | Some concerns |
| **Amitriptyline Vs. Trazodone** | 0.00 | 30.10 | Substantial contribution from bias favouring Trazodone |  | 1.41 (1.07, 1.85) | 1.42 (1.02, 1.95) | No evidence of small-study effects | Some concerns |
| **Amitriptyline Vs. Venlafaxine** | 0.00 | 43.97 | Substantial contribution from bias favouring Venlafaxine |  | 1.06 (0.87, 1.27) | 1.1 (0.87, 1.38) | No evidence of small-study effects | Some concerns |
| **Bupropion Vs. Fluoxetine** | 0.00 | 4.10 | No substantial contribution from bias |  | 1.37 (0.96, 1.96) | 1.39 (0.95, 2.05) | No evidence of small-study effects | Low risk |
| **Bupropion Vs. Paroxetine** | 0.00 | 0.00 | No substantial contribution from bias |  | 1.15 (0.8, 1.67) | 1.19 (0.81, 1.76) | No evidence of small-study effects | Low risk |
| **Bupropion Vs. Sertraline** | 0.00 | 22.15 | Substantial contribution from bias favouring Sertraline |  | 1.23 (0.84, 1.8) | 1.2 (0.81, 1.81) | No evidence of small-study effects | Some concerns |
| **Bupropion Vs. Trazodone** | 0.00 | 30.58 | Substantial contribution from bias favouring Trazodone |  | 1.54 (1.03, 2.31) | 1.53 (0.97, 2.4) | No evidence of small-study effects | Some concerns |
| **Bupropion Vs. Venlafaxine** | 0.00 | 59.25 | Substantial contribution from bias favouring Venlafaxine |  | 1.16 (0.81, 1.66) | 1.18 (0.81, 1.74) | No evidence of small-study effects | Some concerns |
| **Citalopram Vs. Clomipramine** | 23.78 | 0.00 | Substantial contribution from bias favouring Citalopram |  | 1.06 (0.83, 1.38) | 0.98 (0.7, 1.34) | No evidence of small-study effects | Some concerns |
| **Citalopram Vs. Escitalopram** | 9.53 | 77.74 | Substantial contribution from bias favouring Escitalopram |  | 0.8 (0.65, 0.97) | 0.77 (0.61, 0.98) | No evidence of small-study effects | Some concerns |
| **Citalopram Vs. Fluoxetine** | 30.23 | 4.53 | Substantial contribution from bias favouring Citalopram |  | 1.06 (0.87, 1.29) | 1.02 (0.82, 1.26) | No evidence of small-study effects | Some concerns |
| **Citalopram Vs. Fluvoxamine** | 24.23 | 6.68 | Substantial contribution from bias favouring Citalopram |  | 1.07 (0.82, 1.39) | 1.03 (0.74, 1.45) | No evidence of small-study effects | Some concerns |
| **Citalopram Vs. Mirtazapine** | 16.42 | 45.62 | Substantial contribution from bias favouring Mirtazapine |  | 0.83 (0.65, 1.06) | 0.78 (0.59, 1.01) | No evidence of small-study effects | Some concerns |
| **Citalopram Vs. Reboxetine** | 12.37 | 46.28 | Substantial contribution from bias favouring Reboxetine |  | 1.28 (0.92, 1.75) | 1.23 (0.86, 1.78) | No evidence of small-study effects | Some concerns |
| **Citalopram Vs. Sertraline** | 36.53 | 13.34 | Substantial contribution from bias favouring Citalopram |  | 0.95 (0.77, 1.19) | 0.88 (0.7, 1.12) | No evidence of small-study effects | Some concerns |
| **Citalopram Vs. Venlafaxine** | 17.07 | 37.98 | Substantial contribution from bias favouring Venlafaxine |  | 0.9 (0.73, 1.11) | 0.87 (0.67, 1.11) | No evidence of small-study effects | Some concerns |
| **Clomipramine Vs. Fluoxetine** | 0.00 | 27.60 | Substantial contribution from bias favouring Fluoxetine |  | 1 (0.82, 1.22) | 1.04 (0.8, 1.38) | No evidence of small-study effects | Some concerns |
| **Clomipramine Vs. Fluvoxamine** | 0.00 | 22.19 | Substantial contribution from bias favouring Fluvoxamine |  | 1.01 (0.76, 1.32) | 1.06 (0.74, 1.53) | No evidence of small-study effects | Some concerns |
| **Clomipramine Vs. Milnacipran** | 0.00 | 51.29 | Substantial contribution from bias favouring Milnacipran |  | 0.9 (0.67, 1.19) | 0.95 (0.67, 1.33) | No evidence of small-study effects | Some concerns |
| **Clomipramine Vs. Paroxetine** | 0.00 | 0.00 | No substantial contribution from bias |  | 0.84 (0.68, 1.02) | 0.9 (0.69, 1.17) | No evidence of small-study effects | Low risk |
| **Clomipramine Vs. Sertraline** | 0.00 | 34.04 | Substantial contribution from bias favouring Sertraline |  | 0.89 (0.71, 1.12) | 0.9 (0.68, 1.22) | No evidence of small-study effects | Some concerns |
| **Clomipramine Vs. Trazodone** | 0.00 | 26.57 | Substantial contribution from bias favouring Trazodone |  | 1.13 (0.81, 1.52) | 1.15 (0.79, 1.65) | No evidence of small-study effects | Some concerns |
| **Clomipramine Vs. Venlafaxine** | 0.00 | 44.85 | Substantial contribution from bias favouring Venlafaxine |  | 0.84 (0.67, 1.06) | 0.89 (0.66, 1.2) | No evidence of small-study effects | Some concerns |
| **Duloxetine Vs. Escitalopram** | 49.74 | 21.08 | Substantial contribution from bias favouring Duloxetine |  | 0.85 (0.67, 1.08) | 0.86 (0.65, 1.18) | No evidence of small-study effects | Some concerns |
| **Duloxetine Vs. Paroxetine** | 53.40 | 0.00 | Substantial contribution from bias favouring Duloxetine |  | 0.96 (0.76, 1.2) | 0.98 (0.76, 1.29) | No evidence of small-study effects | Some concerns |
| **Duloxetine Vs. Venlafaxine** | 49.14 | 17.37 | Substantial contribution from bias favouring Duloxetine |  | 0.96 (0.77, 1.21) | 0.97 (0.74, 1.29) | No evidence of small-study effects | Some concerns |
| **Escitalopram Vs. Fluoxetine** | 45.08 | 3.66 | Substantial contribution from bias favouring Escitalopram |  | 1.33 (1.11, 1.6) | 1.32 (1.06, 1.63) | No evidence of small-study effects | Some concerns |
| **Escitalopram Vs. Paroxetine** | 42.83 | 0.00 | Substantial contribution from bias favouring Escitalopram |  | 1.12 (0.93, 1.34) | 1.13 (0.91, 1.4) | No evidence of small-study effects | Some concerns |
| **Escitalopram Vs. Sertraline** | 51.11 | 12.28 | Substantial contribution from bias favouring Escitalopram |  | 1.2 (0.96, 1.47) | 1.14 (0.9, 1.45) | No evidence of small-study effects | Some concerns |
| **Escitalopram Vs. Venlafaxine** | 43.86 | 26.99 | Substantial contribution from bias favouring Escitalopram |  | 1.13 (0.92, 1.37) | 1.12 (0.88, 1.42) | No evidence of small-study effects | Some concerns |
| **Fluoxetine Vs. Fluvoxamine** | 27.12 | 10.24 | Substantial contribution from bias favouring Fluoxetine |  | 1.01 (0.81, 1.26) | 1.01 (0.76, 1.35) | No evidence of small-study effects | Some concerns |
| **Fluoxetine Vs. Milnacipran** | 12.29 | 53.29 | Substantial contribution from bias favouring Milnacipran |  | 0.9 (0.7, 1.13) | 0.91 (0.71, 1.16) | No evidence of small-study effects | Some concerns |
| **Fluoxetine Vs. Mirtazapine** | 5.92 | 51.83 | Substantial contribution from bias favouring Mirtazapine |  | 0.78 (0.64, 0.94) | 0.76 (0.62, 0.94) | No evidence of small-study effects | Some concerns |
| **Fluoxetine Vs. Nefazodone** | 4.59 | 56.53 | Substantial contribution from bias favouring Nefazodone |  | 0.96 (0.66, 1.4) | 0.94 (0.59, 1.51) | No evidence of small-study effects | Some concerns |
| **Fluoxetine Vs. Paroxetine** | 10.03 | 0.00 | No substantial contribution from bias |  | 0.84 (0.74, 0.96) | 0.86 (0.74, 0.99) | No evidence of small-study effects | Low risk |
| **Fluoxetine Vs. Reboxetine** | 3.18 | 54.99 | Substantial contribution from bias favouring Reboxetine |  | 1.2 (0.88, 1.63) | 1.21 (0.86, 1.71) | No evidence of small-study effects | Some concerns |
| **Fluoxetine Vs. Sertraline** | 8.52 | 39.61 | Substantial contribution from bias favouring Sertraline |  | 0.9 (0.76, 1.06) | 0.87 (0.73, 1.03) | No evidence of small-study effects | Some concerns |
| **Fluoxetine Vs. Trazodone** | 9.77 | 28.50 | Substantial contribution from bias favouring Trazodone |  | 1.13 (0.86, 1.46) | 1.1 (0.82, 1.47) | No evidence of small-study effects | Some concerns |
| **Fluoxetine Vs. Venlafaxine** | 5.80 | 61.26 | Substantial contribution from bias favouring Venlafaxine |  | 0.85 (0.73, 0.97) | 0.85 (0.71, 1.01) | No evidence of small-study effects | Some concerns |
| **Fluvoxamine Vs. Milnacipran** | 10.19 | 55.81 | Substantial contribution from bias favouring Milnacipran |  | 0.89 (0.66, 1.18) | 0.89 (0.63, 1.25) | No evidence of small-study effects | Some concerns |
| **Fluvoxamine Vs. Mirtazapine** | 6.15 | 56.01 | Substantial contribution from bias favouring Mirtazapine |  | 0.77 (0.6, 1) | 0.75 (0.55, 1.02) | No evidence of small-study effects | Some concerns |
| **Fluvoxamine Vs. Paroxetine** | 11.67 | 0.00 | No substantial contribution from bias |  | 0.84 (0.67, 1.04) | 0.85 (0.63, 1.13) | No evidence of small-study effects | Low risk |
| **Fluvoxamine Vs. Sertraline** | 10.03 | 34.03 | Substantial contribution from bias favouring Sertraline |  | 0.89 (0.7, 1.14) | 0.86 (0.63, 1.16) | No evidence of small-study effects | Some concerns |
| **Fluvoxamine Vs. Venlafaxine** | 9.24 | 40.05 | Substantial contribution from bias favouring Venlafaxine |  | 0.84 (0.66, 1.07) | 0.84 (0.61, 1.14) | No evidence of small-study effects | Some concerns |
| **Milnacipran Vs. Paroxetine** | 65.26 | 0.00 | Substantial contribution from bias favouring Milnacipran |  | 0.94 (0.75, 1.18) | 0.95 (0.74, 1.21) | No evidence of small-study effects | Some concerns |
| **Milnacipran Vs. Sertraline** | 41.50 | 24.27 | Substantial contribution from bias favouring Milnacipran |  | 1 (0.77, 1.3) | 0.96 (0.73, 1.26) | No evidence of small-study effects | Some concerns |
| **Mirtazapine Vs. Paroxetine** | 52.73 | 0.00 | Substantial contribution from bias favouring Mirtazapine |  | 1.08 (0.9, 1.3) | 1.13 (0.91, 1.39) | No evidence of small-study effects | Some concerns |
| **Mirtazapine Vs. Sertraline** | 46.60 | 18.64 | Substantial contribution from bias favouring Mirtazapine |  | 1.15 (0.93, 1.43) | 1.14 (0.89, 1.45) | No evidence of small-study effects | Some concerns |
| **Mirtazapine Vs. Trazodone** | 45.99 | 13.77 | Substantial contribution from bias favouring Mirtazapine |  | 1.45 (1.08, 1.93) | 1.44 (1.04, 2) | No evidence of small-study effects | Some concerns |
| **Mirtazapine Vs. Venlafaxine** | 34.34 | 46.70 | Substantial contribution from bias balanced |  | 1.08 (0.88, 1.32) | 1.11 (0.88, 1.41) | No evidence of small-study effects | Low risk |
| **Nefazodone Vs. Paroxetine** | 67.24 | 0.00 | Substantial contribution from bias favouring Nefazodone |  | 0.88 (0.6, 1.28) | 0.91 (0.57, 1.45) | No evidence of small-study effects | Some concerns |
| **Nefazodone Vs. Sertraline** | 54.83 | 14.69 | Substantial contribution from bias favouring Nefazodone |  | 0.93 (0.63, 1.38) | 0.92 (0.57, 1.48) | No evidence of small-study effects | Some concerns |
| **Paroxetine Vs. Reboxetine** | 0.00 | 47.01 | Substantial contribution from bias favouring Reboxetine |  | 1.43 (1.05, 1.95) | 1.41 (1, 2) | No evidence of small-study effects | Some concerns |
| **Paroxetine Vs. Sertraline** | 0.00 | 21.54 | Substantial contribution from bias favouring Sertraline |  | 1.06 (0.91, 1.26) | 1.01 (0.84, 1.21) | No evidence of small-study effects | Some concerns |
| **Paroxetine Vs. Trazodone** | 0.00 | 19.20 | Substantial contribution from bias favouring Trazodone |  | 1.34 (1.03, 1.73) | 1.28 (0.95, 1.72) | No evidence of small-study effects | Some concerns |
| **Paroxetine Vs. Venlafaxine** | 0.00 | 41.77 | Substantial contribution from bias favouring Venlafaxine |  | 1 (0.86, 1.17) | 0.99 (0.82, 1.19) | No evidence of small-study effects | Some concerns |
| **Reboxetine Vs. Venlafaxine** | 32.69 | 46.58 | Substantial contribution from bias balanced |  | 0.7 (0.52, 0.96) | 0.7 (0.48, 1.01) | No evidence of small-study effects | Low risk |
| **Sertraline Vs. Trazodone** | 32.22 | 18.27 | Substantial contribution from bias balanced |  | 1.26 (0.95, 1.65) | 1.27 (0.92, 1.74) | No evidence of small-study effects | Low risk |
| **Sertraline Vs. Venlafaxine** | 19.77 | 47.68 | Substantial contribution from bias favouring Venlafaxine |  | 0.94 (0.79, 1.13) | 0.98 (0.79, 1.21) | No evidence of small-study effects | Some concerns |
| **Trazodone Vs. Venlafaxine** | 18.21 | 44.02 | Substantial contribution from bias favouring Venlafaxine |  | 0.75 (0.57, 0.98) | 0.77 (0.56, 1.06) | No evidence of small-study effects | Some concerns |
| **Venlafaxine Vs. Vortioxetine** | 0.00 | 0.00 | No substantial contribution from bias |  | 0.72 (0.43, 1.19) | 0.71 (0.43, 1.19) | No evidence of small-study effects | Low risk |
| **Only indirect** | | | | | | | | |
| **Amitriptyline Vs. Bupropion** | 0.00 | 0.00 | No substantial contribution from bias | No bias detected | 0.91 (0.62, 1.33) | 0.93 (0.61, 1.39) | No evidence of small-study effects | Low risk |
| **Amitriptyline Vs. Nefazodone** | 0.00 | 45.16 | Substantial contribution from bias favouring Nefazodone | Suspected bias favouring Nefazodone | 1.2 (0.81, 1.78) | 1.22 (0.74, 1.99) | No evidence of small-study effects | High risk |
| **Agomelatine Vs. Amitriptyline** | 0.00 | 0.00 | No substantial contribution from bias | No bias detected | 0.96 (0.75, 1.22) | 0.91 (0.68, 1.2) | No evidence of small-study effects | Low risk |
| **Agomelatine Vs. Bupropion** | 0.00 | 0.00 | No substantial contribution from bias | No bias detected | 0.87 (0.58, 1.3) | 0.85 (0.55, 1.3) | No evidence of small-study effects | Low risk |
| **Agomelatine Vs. Citalopram** | 0.00 | 13.86 | No substantial contribution from bias | No bias detected | 1.13 (0.87, 1.47) | 1.15 (0.86, 1.54) | No evidence of small-study effects | Low risk |
| **Agomelatine Vs. Clomipramine** | 0.00 | 0.00 | No substantial contribution from bias | No bias detected | 1.2 (0.92, 1.57) | 1.12 (0.8, 1.57) | No evidence of small-study effects | Low risk |
| **Agomelatine Vs. Fluvoxamine** | 0.00 | 7.11 | No substantial contribution from bias | No bias detected | 1.2 (0.91, 1.61) | 1.19 (0.83, 1.69) | No evidence of small-study effects | Low risk |
| **Agomelatine Vs. Milnacipran** | 0.00 | 37.32 | Substantial contribution from bias favouring Milnacipran | No bias detected | 1.07 (0.8, 1.45) | 1.06 (0.77, 1.47) | No evidence of small-study effects | Some concerns |
| **Agomelatine Vs. Mirtazapine** | 0.00 | 34.39 | Substantial contribution from bias favouring Mirtazapine | No bias detected | 0.93 (0.72, 1.21) | 0.89 (0.67, 1.19) | No evidence of small-study effects | Some concerns |
| **Agomelatine Vs. Nefazodone** | 0.00 | 39.26 | Substantial contribution from bias favouring Nefazodone | No bias detected | 1.15 (0.76, 1.74) | 1.11 (0.67, 1.84) | No evidence of small-study effects | Some concerns |
| **Agomelatine Vs. Reboxetine** | 0.00 | 34.34 | Substantial contribution from bias favouring Reboxetine | No bias detected | 1.44 (1.02, 2.05) | 1.42 (0.96, 2.13) | No evidence of small-study effects | Some concerns |
| **Agomelatine Vs. Sertraline** | 0.00 | 17.88 | Substantial contribution from bias favouring Sertraline | No bias detected | 1.07 (0.85, 1.37) | 1.02 (0.78, 1.33) | No evidence of small-study effects | Some concerns |
| **Agomelatine Vs. Trazodone** | 0.00 | 15.08 | Substantial contribution from bias favouring Trazodone | No bias detected | 1.35 (0.98, 1.86) | 1.29 (0.9, 1.84) | No evidence of small-study effects | Some concerns |
| **Agomelatine Vs. Vortioxetine** | 0.00 | 0.00 | No substantial contribution from bias | No bias detected | 0.72 (0.42, 1.25) | 0.71 (0.4, 1.25) | No evidence of small-study effects | Low risk |
| **Amitriptyline Vs. Citalopram** | 0.00 | 19.30 | Substantial contribution from bias favouring Citalopram | Suspected bias favouring Citalopram | 1.18 (0.93, 1.48) | 1.27 (0.97, 1.67) | No evidence of small-study effects | High risk |
| **Amitriptyline Vs. Clomipramine** | 0.00 | 0.00 | No substantial contribution from bias | No bias detected | 1.25 (0.99, 1.59) | 1.24 (0.91, 1.67) | No evidence of small-study effects | Low risk |
| **Amitriptyline Vs. Duloxetine** | 0.00 | 33.46 | Substantial contribution from bias favouring Duloxetine | Suspected bias favouring Duloxetine | 1.1 (0.85, 1.42) | 1.13 (0.82, 1.52) | No evidence of small-study effects | High risk |
| **Amitriptyline Vs. Escitalopram** | 0.00 | 34.23 | Substantial contribution from bias favouring Escitalopram | Suspected bias favouring Escitalopram | 0.94 (0.74, 1.18) | 0.98 (0.75, 1.27) | No evidence of small-study effects | High risk |
| **Amitriptyline Vs. Mirtazapine** | 0.00 | 39.83 | Substantial contribution from bias favouring Mirtazapine | Suspected bias favouring Mirtazapine | 0.98 (0.78, 1.21) | 0.98 (0.76, 1.27) | No evidence of small-study effects | High risk |
| **Amitriptyline Vs. Reboxetine** | 0.00 | 35.24 | Substantial contribution from bias favouring Reboxetine | Suspected bias favouring Reboxetine | 1.5 (1.07, 2.09) | 1.56 (1.07, 2.29) | No evidence of small-study effects | High risk |
| **Amitriptyline Vs. Vortioxetine** | 0.00 | 0.00 | No substantial contribution from bias | No bias detected | 0.76 (0.44, 1.28) | 0.78 (0.44, 1.35) | No evidence of small-study effects | Low risk |
| **Bupropion Vs. Citalopram** | 0.00 | 15.87 | Substantial contribution from bias favouring Citalopram | Suspected bias favouring Citalopram | 1.29 (0.88, 1.93) | 1.36 (0.89, 2.09) | No evidence of small-study effects | High risk |
| **Bupropion Vs. Clomipramine** | 0.00 | 0.00 | No substantial contribution from bias | No bias detected | 1.38 (0.92, 2.07) | 1.33 (0.85, 2.09) | No evidence of small-study effects | Low risk |
| **Bupropion Vs. Duloxetine** | 0.00 | 33.99 | Substantial contribution from bias favouring Duloxetine | Suspected bias favouring Duloxetine | 1.21 (0.81, 1.82) | 1.21 (0.78, 1.9) | No evidence of small-study effects | High risk |
| **Bupropion Vs. Escitalopram** | 0.00 | 32.49 | Substantial contribution from bias favouring Escitalopram | Suspected bias favouring Escitalopram | 1.03 (0.7, 1.53) | 1.05 (0.7, 1.61) | No evidence of small-study effects | High risk |
| **Bupropion Vs. Fluvoxamine** | 0.00 | 7.41 | No substantial contribution from bias | Suspected bias favouring Fluvoxamine | 1.38 (0.92, 2.08) | 1.41 (0.89, 2.25) | No evidence of small-study effects | Low risk |
| **Bupropion Vs. Milnacipran** | 0.00 | 37.49 | Substantial contribution from bias favouring Milnacipran | Suspected bias favouring Milnacipran | 1.23 (0.81, 1.88) | 1.26 (0.82, 1.97) | No evidence of small-study effects | High risk |
| **Bupropion Vs. Mirtazapine** | 0.00 | 35.52 | Substantial contribution from bias favouring Mirtazapine | Suspected bias favouring Mirtazapine | 1.07 (0.72, 1.59) | 1.06 (0.7, 1.61) | No evidence of small-study effects | High risk |
| **Bupropion Vs. Nefazodone** | 0.00 | 38.92 | Substantial contribution from bias favouring Nefazodone | Suspected bias favouring Nefazodone | 1.32 (0.8, 2.23) | 1.31 (0.73, 2.39) | No evidence of small-study effects | High risk |
| **Bupropion Vs. Reboxetine** | 0.00 | 33.10 | Substantial contribution from bias favouring Reboxetine | Suspected bias favouring Reboxetine | 1.65 (1.03, 2.64) | 1.68 (1.02, 2.79) | No evidence of small-study effects | High risk |
| **Bupropion Vs. Vortioxetine** | 0.00 | 0.00 | No substantial contribution from bias | No bias detected | 0.83 (0.45, 1.53) | 0.84 (0.45, 1.61) | No evidence of small-study effects | Low risk |
| **Citalopram Vs. Duloxetine** | 10.33 | 32.46 | Substantial contribution from bias favouring Duloxetine | Suspected bias favouring Duloxetine | 0.93 (0.71, 1.23) | 0.89 (0.64, 1.21) | No evidence of small-study effects | High risk |
| **Citalopram Vs. Milnacipran** | 17.42 | 34.04 | Substantial contribution from bias favouring Milnacipran | Suspected bias favouring Milnacipran | 0.95 (0.72, 1.26) | 0.92 (0.67, 1.25) | No evidence of small-study effects | High risk |
| **Citalopram Vs. Nefazodone** | 19.65 | 36.88 | Substantial contribution from bias favouring Nefazodone | Suspected bias favouring Nefazodone | 1.02 (0.67, 1.55) | 0.96 (0.58, 1.6) | No evidence of small-study effects | High risk |
| **Citalopram Vs. Paroxetine** | 17.53 | 0.00 | Substantial contribution from bias favouring Citalopram | No bias detected | 0.89 (0.73, 1.1) | 0.88 (0.69, 1.1) | No evidence of small-study effects | Some concerns |
| **Citalopram Vs. Trazodone** | 18.15 | 14.79 | Substantial contribution from bias balanced | Suspected bias favouring Trazodone | 1.19 (0.88, 1.63) | 1.12 (0.78, 1.58) | No evidence of small-study effects | Low risk |
| **Citalopram Vs. Vortioxetine** | 11.59 | 0.00 | No substantial contribution from bias | No bias detected | 0.64 (0.37, 1.12) | 0.62 (0.35, 1.07) | No evidence of small-study effects | Low risk |
| **Clomipramine Vs. Duloxetine** | 0.00 | 34.20 | Substantial contribution from bias favouring Duloxetine | Suspected bias favouring Duloxetine | 0.88 (0.66, 1.16) | 0.91 (0.63, 1.29) | No evidence of small-study effects | High risk |
| **Clomipramine Vs. Escitalopram** | 0.00 | 35.02 | Substantial contribution from bias favouring Escitalopram | Suspected bias favouring Escitalopram | 0.75 (0.58, 0.96) | 0.79 (0.58, 1.09) | No evidence of small-study effects | High risk |
| **Clomipramine Vs. Mirtazapine** | 0.00 | 40.40 | Substantial contribution from bias favouring Mirtazapine | Suspected bias favouring Mirtazapine | 0.78 (0.6, 1.01) | 0.79 (0.58, 1.1) | No evidence of small-study effects | High risk |
| **Clomipramine Vs. Nefazodone** | 0.00 | 43.59 | Substantial contribution from bias favouring Nefazodone | Suspected bias favouring Nefazodone | 0.96 (0.63, 1.47) | 0.99 (0.59, 1.65) | No evidence of small-study effects | High risk |
| **Clomipramine Vs. Reboxetine** | 0.00 | 34.10 | Substantial contribution from bias favouring Reboxetine | Suspected bias favouring Reboxetine | 1.2 (0.84, 1.72) | 1.27 (0.83, 1.94) | No evidence of small-study effects | High risk |
| **Clomipramine Vs. Vortioxetine** | 0.00 | 0.00 | No substantial contribution from bias | No bias detected | 0.6 (0.34, 1.04) | 0.63 (0.35, 1.13) | No evidence of small-study effects | Low risk |
| **Duloxetine Vs. Fluoxetine** | 34.34 | 4.70 | Substantial contribution from bias favouring Duloxetine | Suspected bias favouring Duloxetine | 1.14 (0.91, 1.44) | 1.14 (0.88, 1.5) | No evidence of small-study effects | High risk |
| **Duloxetine Vs. Fluvoxamine** | 28.26 | 7.24 | Substantial contribution from bias favouring Duloxetine | Suspected bias favouring Duloxetine | 1.14 (0.85, 1.55) | 1.16 (0.81, 1.7) | No evidence of small-study effects | High risk |
| **Duloxetine Vs. Milnacipran** | 30.60 | 35.51 | Substantial contribution from bias balanced | Suspected bias favouring Duloxetine | 1.02 (0.75, 1.4) | 1.03 (0.74, 1.48) | No evidence of small-study effects | Low risk |
| **Duloxetine Vs. Mirtazapine** | 33.66 | 30.97 | Substantial contribution from bias balanced | Suspected bias favouring Duloxetine | 0.89 (0.67, 1.17) | 0.87 (0.64, 1.2) | No evidence of small-study effects | Low risk |
| **Duloxetine Vs. Nefazodone** | 32.14 | 37.16 | Substantial contribution from bias balanced | Suspected bias favouring Duloxetine | 1.09 (0.71, 1.68) | 1.08 (0.64, 1.83) | No evidence of small-study effects | Low risk |
| **Duloxetine Vs. Reboxetine** | 34.10 | 29.65 | Substantial contribution from bias balanced | Suspected bias favouring Duloxetine | 1.37 (0.94, 1.97) | 1.39 (0.92, 2.1) | No evidence of small-study effects | Low risk |
| **Duloxetine Vs. Sertraline** | 33.88 | 13.45 | Substantial contribution from bias favouring Duloxetine | Suspected bias favouring Duloxetine | 1.02 (0.79, 1.31) | 0.99 (0.75, 1.35) | No evidence of small-study effects | High risk |
| **Duloxetine Vs. Trazodone** | 32.28 | 12.06 | Substantial contribution from bias favouring Duloxetine | Suspected bias favouring Duloxetine | 1.28 (0.92, 1.78) | 1.26 (0.86, 1.85) | No evidence of small-study effects | High risk |
| **Duloxetine Vs. Vortioxetine** | 28.91 | 0.00 | Substantial contribution from bias favouring Duloxetine | No bias detected | 0.69 (0.39, 1.2) | 0.69 (0.39, 1.24) | No evidence of small-study effects | Some concerns |
| **Escitalopram Vs. Fluvoxamine** | 33.32 | 6.52 | Substantial contribution from bias favouring Escitalopram | Suspected bias favouring Escitalopram | 1.34 (1.02, 1.75) | 1.34 (0.96, 1.87) | No evidence of small-study effects | High risk |
| **Escitalopram Vs. Milnacipran** | 29.80 | 35.11 | Substantial contribution from bias balanced | Suspected bias favouring Escitalopram | 1.2 (0.91, 1.58) | 1.2 (0.88, 1.63) | No evidence of small-study effects | Low risk |
| **Escitalopram Vs. Mirtazapine** | 37.14 | 35.93 | Substantial contribution from bias balanced | Suspected bias favouring Escitalopram | 1.04 (0.81, 1.33) | 1.01 (0.77, 1.31) | No evidence of small-study effects | Low risk |
| **Escitalopram Vs. Nefazodone** | 33.61 | 38.75 | Substantial contribution from bias balanced | Suspected bias favouring Escitalopram | 1.28 (0.84, 1.92) | 1.24 (0.76, 2.06) | No evidence of small-study effects | Low risk |
| **Escitalopram Vs. Reboxetine** | 37.07 | 34.58 | Substantial contribution from bias balanced | Suspected bias favouring Escitalopram | 1.61 (1.15, 2.22) | 1.6 (1.09, 2.34) | No evidence of small-study effects | Low risk |
| **Escitalopram Vs. Trazodone** | 33.55 | 13.64 | Substantial contribution from bias favouring Escitalopram | Suspected bias favouring Escitalopram | 1.51 (1.1, 2.03) | 1.45 (1.03, 2.04) | No evidence of small-study effects | High risk |
| **Escitalopram Vs. Vortioxetine** | 27.19 | 0.00 | Substantial contribution from bias favouring Escitalopram | No bias detected | 0.81 (0.46, 1.39) | 0.8 (0.45, 1.38) | No evidence of small-study effects | Some concerns |
| **Fluoxetine Vs. Vortioxetine** | 3.97 | 0.00 | No substantial contribution from bias | No bias detected | 0.6 (0.35, 1.01) | 0.61 (0.35, 1.03) | No evidence of small-study effects | Low risk |
| **Fluvoxamine Vs. Nefazodone** | 7.62 | 38.15 | Substantial contribution from bias favouring Nefazodone | Suspected bias favouring Nefazodone | 0.95 (0.62, 1.46) | 0.93 (0.55, 1.58) | No evidence of small-study effects | High risk |
| **Fluvoxamine Vs. Reboxetine** | 7.57 | 31.53 | Substantial contribution from bias favouring Reboxetine | Suspected bias favouring Reboxetine | 1.2 (0.83, 1.7) | 1.2 (0.77, 1.83) | No evidence of small-study effects | High risk |
| **Fluvoxamine Vs. Trazodone** | 11.64 | 19.55 | Substantial contribution from bias balanced | Suspected bias favouring Fluvoxamine | 1.12 (0.81, 1.54) | 1.09 (0.73, 1.6) | No evidence of small-study effects | Low risk |
| **Fluvoxamine Vs. Vortioxetine** | 6.50 | 0.00 | No substantial contribution from bias | No bias detected | 0.6 (0.34, 1.05) | 0.6 (0.33, 1.07) | No evidence of small-study effects | Low risk |
| **Milnacipran Vs. Mirtazapine** | 41.56 | 38.12 | Substantial contribution from bias balanced | Suspected bias favouring Milnacipran | 0.87 (0.66, 1.15) | 0.84 (0.62, 1.13) | No evidence of small-study effects | Low risk |
| **Milnacipran Vs. Nefazodone** | 41.49 | 41.49 | Substantial contribution from bias balanced | Suspected bias favouring Milnacipran | 1.07 (0.7, 1.63) | 1.04 (0.62, 1.75) | No evidence of small-study effects | Low risk |
| **Milnacipran Vs. Reboxetine** | 37.88 | 32.84 | Substantial contribution from bias balanced | Suspected bias favouring Reboxetine | 1.34 (0.93, 1.93) | 1.34 (0.89, 2.02) | No evidence of small-study effects | Low risk |
| **Milnacipran Vs. Trazodone** | 43.26 | 20.28 | Substantial contribution from bias favouring Milnacipran | Suspected bias favouring Trazodone | 1.26 (0.9, 1.74) | 1.21 (0.83, 1.75) | No evidence of small-study effects | Some concerns |
| **Milnacipran Vs. Venlafaxine** | 39.54 | 33.78 | Substantial contribution from bias balanced | Suspected bias favouring Venlafaxine | 0.94 (0.73, 1.21) | 0.94 (0.7, 1.25) | No evidence of small-study effects | Low risk |
| **Milnacipran Vs. Vortioxetine** | 27.96 | 0.00 | Substantial contribution from bias favouring Milnacipran | No bias detected | 0.67 (0.38, 1.17) | 0.67 (0.37, 1.19) | No evidence of small-study effects | Some concerns |
| **Mirtazapine Vs. Nefazodone** | 37.50 | 41.22 | Substantial contribution from bias balanced | Suspected bias favouring Nefazodone | 1.23 (0.81, 1.85) | 1.24 (0.76, 2.04) | No evidence of small-study effects | Low risk |
| **Mirtazapine Vs. Reboxetine** | 37.70 | 36.38 | Substantial contribution from bias balanced | Suspected bias favouring Reboxetine | 1.54 (1.09, 2.17) | 1.59 (1.08, 2.35) | No evidence of small-study effects | Low risk |
| **Mirtazapine Vs. Vortioxetine** | 23.93 | 0.00 | Substantial contribution from bias favouring Mirtazapine | No bias detected | 0.77 (0.45, 1.33) | 0.8 (0.45, 1.39) | No evidence of small-study effects | Some concerns |
| **Nefazodone Vs. Reboxetine** | 39.74 | 34.99 | Substantial contribution from bias balanced | Suspected bias favouring Reboxetine | 1.25 (0.79, 2) | 1.29 (0.72, 2.28) | No evidence of small-study effects | Low risk |
| **Nefazodone Vs. Trazodone** | 41.23 | 15.79 | Substantial contribution from bias favouring Nefazodone | Suspected bias favouring Nefazodone | 1.17 (0.75, 1.85) | 1.17 (0.68, 1.98) | No evidence of small-study effects | High risk |
| **Nefazodone Vs. Venlafaxine** | 40.54 | 34.41 | Substantial contribution from bias balanced | Suspected bias favouring Venlafaxine | 0.88 (0.59, 1.31) | 0.9 (0.56, 1.46) | No evidence of small-study effects | Low risk |
| **Nefazodone Vs. Vortioxetine** | 28.50 | 0.00 | Substantial contribution from bias favouring Nefazodone | No bias detected | 0.63 (0.33, 1.2) | 0.64 (0.32, 1.29) | No evidence of small-study effects | Some concerns |
| **Paroxetine Vs. Vortioxetine** | 0.00 | 0.00 | No substantial contribution from bias | No bias detected | 0.72 (0.42, 1.21) | 0.71 (0.41, 1.2) | No evidence of small-study effects | Low risk |
| **Reboxetine Vs. Sertraline** | 35.19 | 18.50 | Substantial contribution from bias favouring Reboxetine | Suspected bias favouring Reboxetine | 0.75 (0.54, 1.04) | 0.72 (0.49, 1.04) | No evidence of small-study effects | High risk |
| **Reboxetine Vs. Trazodone** | 32.36 | 15.20 | Substantial contribution from bias favouring Reboxetine | Suspected bias favouring Reboxetine | 0.94 (0.63, 1.39) | 0.91 (0.58, 1.41) | No evidence of small-study effects | High risk |
| **Reboxetine Vs. Vortioxetine** | 22.99 | 0.00 | Substantial contribution from bias favouring Reboxetine | No bias detected | 0.5 (0.27, 0.92) | 0.5 (0.27, 0.93) | No evidence of small-study effects | Some concerns |
| **Sertraline Vs. Vortioxetine** | 13.42 | 0.00 | No substantial contribution from bias | No bias detected | 0.67 (0.39, 1.15) | 0.7 (0.4, 1.2) | No evidence of small-study effects | Low risk |
| **Trazodone Vs. Vortioxetine** | 12.50 | 0.00 | No substantial contribution from bias | No bias detected | 0.53 (0.3, 0.95) | 0.55 (0.3, 1) | No evidence of small-study effects | Low risk |
